# Automated evaluation of accessibility issues of webpage content: tool and evaluation

**DOI:** 10.1038/s41598-025-92192-5

**Published:** 2025-03-19

**Authors:** Jinat Ara, Cecilia Sik-Lanyi

**Affiliations:** 1 Department of Electrical Engineering and Information Systems, University of Pannonia, Egyetem u. 10, Veszprem, 8200 Hungary; 2 Hungarian Research Network, Budapest, Hungary

**Keywords:** Web accessibility issues, Structural and visual elements, Semantic and non-semantic perspective, Algorithmic evaluation, Automated tools

## Abstract

In recent years, there has been a growing field of research focused on comprehending complexity in relation to web platform accessibility. It has shown that it is quite difficult to accurately assess and identify web accessibility concerns while taking multifaceted factors into account. It is imperative to prioritize multi-dimensional characteristics as they facilitate the integration of many aspects into the assessment process, which is a critical component in enhancing the accessibility evaluation process. Although many existing solutions with varying degrees of computational success have been proposed by scholars, they are confined to (1) following a certain set of rules of a specific guideline; (2) limited evaluation properties; (3) disregard for user criteria; and (4) complex functional properties or architectural design. To address these problems, we present in this work a straightforward yet precise model that assesses webpage accessibility by taking into account common features of the structural and visual elements of webpages that are part of the HTML Document Object Model (DOM) structure. In order to predict a webpage’s accessibility status, we implemented three distinct algorithms to analyze web features/objects considering both semantic and non-semantic aspects. We performed experimental work to validate 20 university webpages in Hungary through our developed tool. The computed result of the developed tool was assessed by comparing the result with a user study where we performed user testing that included 40 users’ 80 reviews on the same 20 university webpages in Hungary. Additionally, we compared our developed tool with other scientific models (that already exist) and existing ten open-source commercial automated testing tools considering several functional characteristics or properties. This two-phase assessment result shows that the developed tool has several advanced properties and the potential to predict the accessibility issues of the tested webpages.

**Supplementary Information:**

The online version contains supplementary material available at 10.1038/s41598-025-92192-5.

## Introduction

Since the pandemic made it clear how crucial it is to make the web platform accessible for the community, there has been an increasing interest in the last few years in understanding the complexity associated with accessibility criteria, particularly for the web platform (i.e., webpages)^1^. Although, in the literature, the complexity of the web platform is often described through webpage accessibility and usability frameworks (framework refers to the general structure, methodology, and theoretical foundations that direct the process of investigation), it is frequently ignored by the modern community^2^. When a web platform is fully accessible, it can be utilized with ease and guarantees that all accessibility requirements are fully integrated during the web design and development process. According to several studies, the majority of webpages nowadays concentrate on interactive design and representation elements including eye-catching captions, vibrant layouts, and intricate pictures to subtly enhance user interaction, understandability, and navigation^3^. Also, some studies^4,5^ using user surveys found that these interactive designs do not guarantee total accessibility for a specific user group with special needs. Besides, some research demonstrated that a wide range of factors, including human cognitive state, visual perception, and content navigation experiences, might impact web accessibility and define various user perspectives for webpage information processing.

Recently, researchers proposed several models (model refers to the representation of structural or mathematical depiction of the process of the system) applying different strategies by addressing how accessibility criteria should be merged or handled from the design phase that could successfully increase the webpages’ accessibility (details are provided in section "Background and related work"). To ameliorate the deficiencies of the current situation, Schiavone and Paterno^6^ discussed the significance of implementing updated versions of Web Content Accessibility Guidelines (WCAG). Others discussed the significance of individualized webpage and dynamic webpages evaluation methods^7^. A few studies discussed the significance of using a variety of requirements or criteria along with standard guidelines in the evaluation process in addition to accepted practices. Furthermore, according to other studies, they proposed several sophisticated systems with a considerable degree of potential for evaluating accessibility while taking development and functionality factors into account^8^. Despite the potential of the current technique, most of the existing models have a certain level of vulnerabilities related to the effectiveness of the evaluated results.

Focusing on this effectiveness issue, in one of our previous studies, we analyzed several existing literatures that are available as web accessibility evaluation techniques or solutions or methods or tools in order to identify their existing limitations that can be found in^9^. This broad review work considered both automated and hybrid solutions to determine the most frequently appeared issues that are leading cause to reduce the effectiveness of the existing solutions. However, this broadened investigation depicts that a number of issues appeared in the existing models that misled the development process of the automated web content accessibility testing tool and made the evaluation results ineffective such as i) applying the wrong guidelines, ii) not comprehending the guidelines logically, iii) not utilizing user and expert recommendations as evaluation criteria, iv) giving insufficient significance of semantic viewpoints, and v) unwillingness to utilize the advanced evaluation techniques.

Therefore, addressing the earlier discussion and following the determined factors from our previous study^9^, in this work, our aim is to develop an updated automated web content accessibility evaluation tool by incorporating advanced engineering techniques that can facilitate the development process and improve the effectiveness of the evaluation result by minimizing the existing shortcomings. To achieve this, we developed our automated tool considering several advanced engineering aspects related to design and development strategies that were normally ignored in the previously developed tools such as:

1. Implemented the latest version of Web Content Accessibility Guideline (WCAG 2.2) with a larger number of success criteria than previously implemented in the existing systems,
2. Considered user and expert-suggested criteria as additional criteria along with the WCAG 2.2,
3. Applied knowledge simplification concept for simplifying the WCAG 2.2 and user and expert suggested criteria to logically encoded into the system,
4. Implemented several auxiliary methods to evaluate textual and non-textual web objects,
5. Focused on the semantic perspective of textual web objects incorporating several NLP techniques and
6. Represented the evaluation report in both textual and graphical format to improve its effectiveness in understanding for the end user.

To achieve our goal, we formalize our development by proposing an algorithmic evaluation process where three different algorithms perform by analyzing the common elements of an HTML Document Object Model (DOM) structure in accordance with standard accessibility guidelines and user and expert-suggested criteria derived from our developed accessibility analysis and evaluation framework in^9^. These algorithms serve as an overall accessibility status indicator for the tested webpages. The entire algorithmic evaluation incorporates several auxiliary methods and advanced Artificial Intelligence techniques such as Natural Language Processing (NLP)^10^. The detail tool design, development, and implementation strategies can be found in Section "Methodology".

Throughout the tool design, development and implementation process, we consider the expert’s suggestion for getting direction about how to deal with additional criteria in the evaluation process, and how to parse each information from the guideline and do the inferencing process with the extracted content from the webpage through algorithmic process. Also, we received detailed instructions on how to structure the evaluation results using several graphical representations that include an accessibility score for each algorithmic process, a score for each type of disability, and an overall accessibility score. These are the most important factors that might improve the effectiveness and understandability of the developed tool.

Furthermore, to evaluate the effectiveness of the developed tool, we performed a user assessment where 40 users participated in which they were asked to rate 20 Hungarian university webpages twice based on their perceptions. We then compared the results of the user ratings with the scores we calculated using the developed tool for the same 20 webpages. For the majority of the evaluated webpages, the accessibility status as determined by the user score and the accessibility status as calculated by the developed tool were comparatively similar. These results indicate that the developed Web Content Accessibility Evaluation Environment (WCAEE) tool is effective in evaluating and computing accessibility scores that align with the perception of the participants. It also indicates that the developed tool has a high level of concern for the complexity or issues associated with the accessibility of a webpage, and it is capable of evaluating accessibility based on the structural elements of the webpages.

Besides, to validate the effectiveness of the developed tool in terms of interactive functional properties, we performed a comparative evaluation where, our developed tool is validated by comparing it with other existing seven scientific models and ten existing automated open-source tools (Accessibility Checker, AccessMonitor, aCe, AChecker, Bulk Accessibility Checker, MAUVE, Rocket Validator, TAW, WAVE, and Nibbler) based on several functional properties. These comparative assessments represent that the developed tool has several interactive functionalities that can facilitate the evaluation report to improve its effectiveness and performance.

However, our developed tool is computationally accurate and can assess the accessibility of a specific webpage. The developed tool can be effective for a wide array of people (i.e., end users, designers, developers, practitioners, etc.) to understand the accessibility issues of the tested webpage. Additionally, the user-suggested criteria make the developed tool stand out or distinguishable from others by allowing the computation of accessibility scores for each type of person with disability with the total accessibility scores.

In conclusion, in the later part of the paper, we provide a thorough explanation of the technologies, methodologies, and techniques that have been implemented along with the design, development, and implementation strategies. Several assessment strategies that are considered to evaluate and validate the developed tool is also presented. However, this paper is structured as follows: Section "Background and related work" discusses similar studies that focused on the development of accessibility evaluation techniques and models with their descriptive analysis. Section "Methodology" explains the research methodology by describing the developed tool with its design, development, and implementation strategies, functionalities, and benefits. Also, in this section, we provide evaluation strategies (considering the comparative evaluation of user assessment and assessment through the developed tool) and validation strategies (considering the functional properties to determine the significance of the developed tool). Section "Discussion" provides a detailed discussion of the development with its benefits, challenges, limitations, and future improvement directions. Finally, Section "Conclusion" outlines the conclusions of this study.

## Background and related work

The process of ensuring the availability of online resources or materials is known as digital accessibility^11,12^. The main goal of digital accessibility is to create an online platform that is operable, interactive, and accessible so that individuals with disabilities have equal access to digital content^13,14^. A wide array of aspects initiates barriers to practical implementation of accessibility criteria to ensure digital platforms as an accessible platform such as limited accessibility knowledge and its guidelines. In order to address these problems and provide an accessible solution (such as an application, website, software, etc.), a number of public and corporate organizations, as well as the governments of several nations have created several accessibility rules and regulations such as Web Content Accessibility Guideline (WCAG). WCAG is a published standard that describes all the accessibility criteria and provides step-by-step recommendations about implementation, improvement, and measurement of accessibility to ensure a better user experience for individuals with impairments. Following a standard guideline, it is possible to overcome accessibility issues to ensure digital accessibility.

The upgrowing inaccessible issue or accessing complexity of internet platforms is comparatively related to the webpages which is particularly referred to by the term webpage accessibility. In actual cases, webpage accessing complexity can be defined as “the degree of difficulty in providing full access of web resources for every group of people including special needs”. With this focus, several approaches have been proposed to determine the complexity of accessibility for people with special needs. However, several researchers claimed that the proposed approaches are not free from limitations. For example, Boyalakuntla et al.^15^ proposed an automated accessibility evaluation tool that offers access through the command line and browser plugin. It supports WCAG 2.1 and WCAG 2.2 standards and displays a list of errors with further repairment suggestions through a snippet of code. Although their approach is effective, assessing the webpages in terms of 16 success criteria from WCAG 2.1, and 2.2 standards limits its effectiveness. Also, without computing an overall accessibility score, and ignorance of additional success criteria might limit the reflection of the actual accessibility scenario of the tested webpages. Besides, Pelzetter^16^ proposed a declarative model to assess webpage accessibility in terms of Accessibility Conformance Testing (ACT) rule set and ontology modeling. Despite the effectiveness of the suggested system, a few possible issues have been noted that might limit how useful the evaluation’s outcome is. For example, ontology modeling introduces uncertainty into the testing procedure and decreases the usefulness of the assessed outcome. Furthermore, it might be challenging for practitioners to execute ACT guidelines since they call for resources and experience that may not be readily available.

Some studies attempted to determine web accessibility of a webpage through user perception^17^ while others studied to determine webpage accessibility through human cognition where human cognition refers to the way of retrieving or processing the information on a webpage by different groups of users^18^. According to both user perception and human cognition perspective, it is concluded that various aspects including the excessive amount of content on the webpage can cause an overload of information on the page that directly affects accessibility issues for a particular group of users.

Generally, evaluating the accessibility perspective of a webpage is a process of extracting and evaluating information about web objects in terms of the criteria and requirements of each group of users. Therefore, depending on the needs of each individual, webpage elements or objects such as patterns, animations, layout, and other design and functioning properties can influence the human cognition process and can directly affect webpage accessibility as the majority of the webpages represent their content with various advanced and fancy layout to make their content more implicit to improve the user navigation, understanding and interactivity of the web content^19^. Research conducted by^20^ shows that interactive components such as sharp layouts, snappy captions, and decorative images have a great effect on transforming monotonous information into interactive ways. However, although the interactive components have a great effect, at the same time, it also needs to be reminded that to improve the interactivity, it shouldn’t hinder accessing opportunities.

However, in recent years, different approaches have been proposed to measure accessibility issues of webpages from different aspects and dimensions^21^. Most of the studies represented approaches mostly considered several webpage elements such as Rich Internet Applications (RIAs) visual elements like links, images, menus, buttons, dropdown menus, etc. To evaluate these elements of webpages, most of the approaches typically used HTML source code that was converted into a Document Object Model (DOM) structure to evaluate a set of web features for measuring accessibility issues or complexity of webpages. Although another study conducted by Freire and Fortes^22^, presented an approach for dynamic webpage evaluation considering XML and XSLT scripting documents.

Some studies also invested the value of arbitrary and non-arbitrary information such as textual information, image content, etc. to provide a clear direction about how to represent them in an effective way. Web platforms are continuously changing with advanced design elements and introducing several advanced tools and techniques to make web platforms more attractive to the community. Thus, one study conducted by^23^, where tried to identify the most effective design criteria to make the web more effective with clear statistics about how those criteria have a great impact on making the web effective. Their findings are related to the frequent use of graphical elements for organizing and designing the information such as images, icons, and bullet points. Even though these graphical components or elements enhance the visual appearance of webpages, they also increase serious accessibility issues for people with cognitive aspects.

Several past studies asserted that accessibility also depends on the visual complexity of a webpage though it is barely considered in the literature^11^. In a recent study, Rojbi et al.^24^ addressed that the majority of the visual elements are not accessible to the end users which marked web accessibility as a challenging task. For example, Michailidou et al.^25^ used a set of web features or elements of the tested webpages (such as Menus, Images, Words, Links, and Top Left Corner) to evaluate the accessibility issues considering visual aspects. They evaluated the source code by taking it as input for the model. Considering the same issue, another example was given by Duarte et al.^26^, where they also used web elements to measure their level of accessibility from visual perspectives. They used two certain features such as menu and list elements and evaluated them in terms of their defined role to check whether their identifiable role can be determined to define their accessibility. In another study, Doush et al.^27^ presented an approach to predict whether or not the given webpage is accessible in terms of specified RIAs as it has a great effect on improving the accessibility of visual components.

Again, referring to the visual complexity, several studies addressed that the process of designing a webpage with a lot of different colors may change how accessible it is for users with color vision problems, such as color perception or differentiation. Bonacin et al.^28^ proposed an ontology-based framework with the goal of improving web accessibility and interactivity for individuals with Color Vision Deficiency (CVD). With this technique, the optimal recoloring interface for CVD users can be determined according to their individual tastes. Additionally, Robal et al.^29^ stated that the user interface of webpages should be created with end-user requirements in mind so that users may browse the structure easily and smoothly and understand the information being displayed to them. They created an automated ontology-based assessment of the webpages user interface (UI) to ascertain its accessibility and usability in order to guarantee these factors. These advancements are useful, but there are certain challenges that have hindered their success, such as the need for practitioners to undertake the laborious processes of adding new guidelines and regular updating in ontology-based solutions. Evaluation results could be misleading, and the user might not find them entirely acceptable without a professional examination of the validated outcome.

Also, researchers showed their concerns regarding the quality of webpages, arguing that in order to meet end users’ expectations, additional care must be taken. They asserted that a webpage quality also indicates how accessible it is. In light of this, Rashida et al.^30^ proposed three algorithms for the evaluation of loading time, overall performance qualities, and content information aspects that are typically overlooked in many approaches. In another study, Alsaeedi^31^ not only addressed the same issue but also proposed an algorithmic evaluation methodology that would use several automated accessibility testing tools to assess a webpages accessibility. It makes it possible to choose evaluation tool sets in advance of the test and to compare the accessibility of an old and new version of a certain webpage. These approaches benefit greatly from algorithmic evaluation, yet these techniques have certain drawbacks that limit their use. For example, assessment features are quite few and other features must be taken into consideration in the evaluation process. A variety of user and expert interventions are needed to validate the algorithmic solution or assessment result. These interventions will also help to increase the end user’s acceptability of the evaluated results.

Nowadays, webpage accessibility-related studies are not limited to considering visual perspectives only. In particular, in a recent work, Duarte et al.^32^ proposed an automated approach that helps to understand the semantic meaning of web elements and their correlation with their provided textual format. It takes visual components, particularly images, and evaluates them to determine the similarity between visual components and their textual description according to web content accessibility guidelines. This could allow us to understand how complicated the presented component is for user understanding.

Further, another study was conducted by^33^ where the authors examined how various structural elements can affect web accessibility. For example, the effect of webpage text and background color combination, and the improper font type and font size have a great influence on reducing readability. In some cases, when structural elements-based features are considered instead of visual components only such as audio and video files, and their description or media alternative, page language, input name, proper role and value, etc., the perception of accessibility understanding has significantly increased which derived in one of the studies conducted by Ikhsan and Candra^34^. Also, some researchers considered structural-based features as more beneficial compared to only visual features as structural elements are mostly specified in the source code of webpages which provides detailed information regarding their structure and visual representation at the same time. For example, studies conducted by Doush et al.^35^ considered several structured elements such as alt text properties and captions for non-text content, CAPTCHA, and Text-input field. They concluded that considering the structural elements is a wiser decision and helps to provide more efficient and reliable assessment results.

As webpage is a combination of a large set of structural and design elements that we refer to as variables such as images, links, text, size, color, tables, menu, etc. Thus, it is significantly important to include every element or object as much as possible in the investigation process. Therefore, in our study, we attempted to incorporate more webpage elements associated with the WCAG guidelines, rather than selective ones and examine their status in terms of accessibility aspects to determine their level of accessibility. However, our proposed tool is straightforward and simple to comprehend, thus, it can provide accurate and reliable accessibility reports and compute accessibility scores based on common aspects of an HTML Document Object Model (DOM) structure from both visual and structural elements or properties. Additionally, our proposed tool is computationally easy to implement and does not require extensive computation time and resources.

## Methodology

The objective of this section is twofold. The first objective is to provide details about the proposed Web Content Accessibility Evaluation Environment (WCAEE) tool design, development, and implementation strategies (can be found in section "WCAEE tool design, development and implementation methodology"). The second objective is to present the evaluation and validation strategies of the developed WCAEE tool considering user assessment and comparative assessment of functional properties to represent its effectiveness and enrichment in accessibility evaluation (can be found in section "WCAEE tool evaluation and validation methodology").

### WCAEE tool design, development and implementation methodology

Referring to the understanding from section "Background and related work", it is becoming clear that a modern and sophisticated web content accessibility testing tool is important to assess webpages to accurately reflect their current accessibility scenario. With this need in consideration, we state that this work aims to develop a Web Content Accessibility Evaluation Environment (WCAEE), an advanced tool for evaluating accessibility of webpage content that can be used as an automatic tool to assess the accessibility of web content and determine its accessibility score. Therefore, in this section, our prime objective is to present the WCAEE tool design, development, and implementation strategies in detail that will help to reflect the potential of the development. The detailed design, development, and implementation process of the proposed tool is described in the following sections, where we presented the design, development, and implemented strategies including structural design, variable identification, algorithmic evaluation, and report formulation that have been followed to develop and implement the proposed model as a tool for real-life experimentation. The developed tool has been addressed as WCAEE throughout the paper.

#### WCAEE tool design

The literature suggested that a number of factors, such as the implementation of appropriate guidelines, the consideration of additional criteria, and the use of advanced engineering techniques could facilitate the development process of automated web content accessibility testing tools^9,36–38^. By considering these aspects, and facilitating the development process, it is crucial to propose an accessibility evaluation framework by considering an appropriate accessibility guideline, incorporating user and expert suggestions, integrating guideline knowledge simplification, and applying advanced techniques to facilitate the appropriate accessibility evaluation score computation of the tested webpages.

**Fig. 1:**
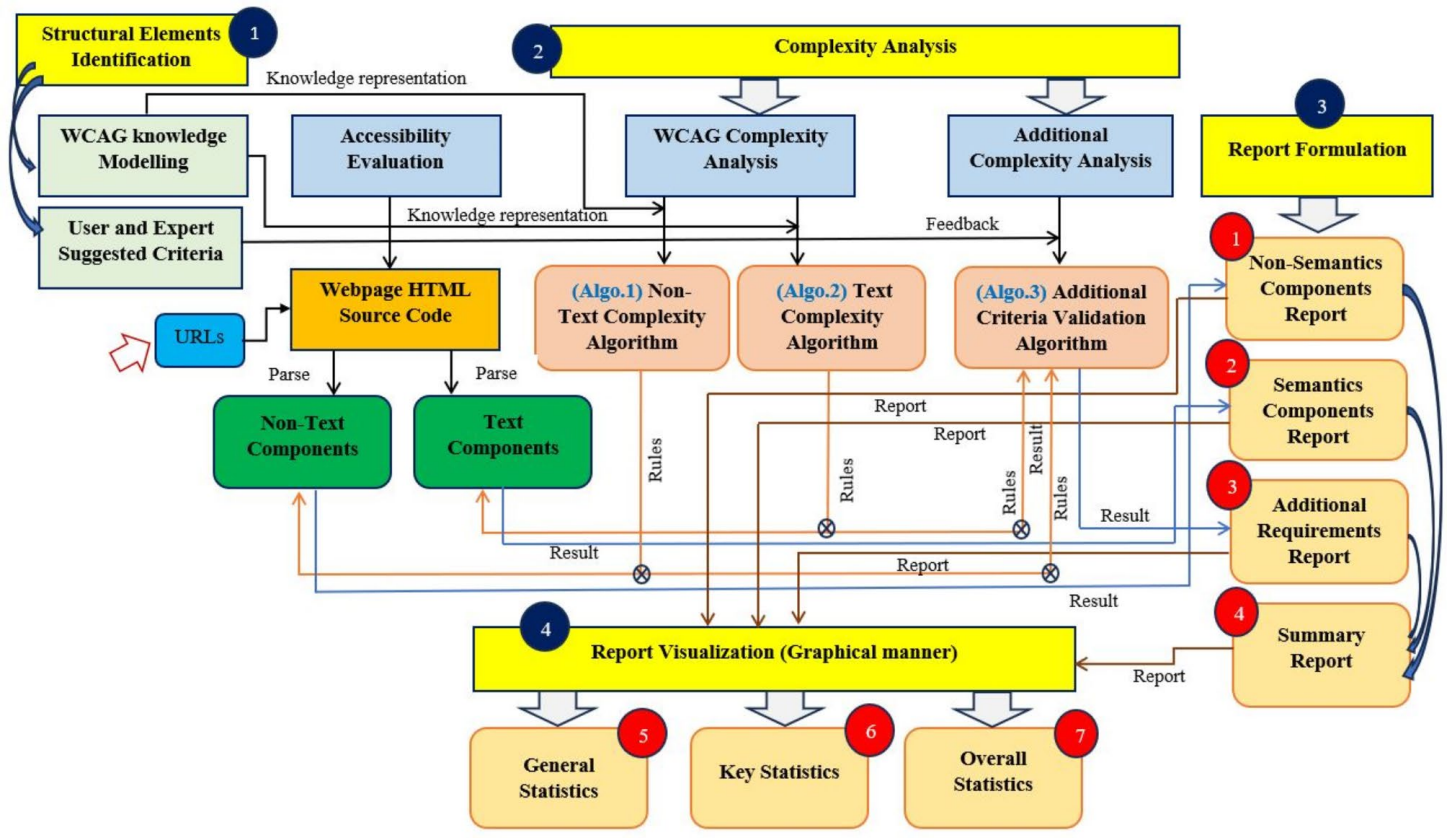
The system structure/views of the developed WCAEE tool.

Figure 1 shows the system architecture of the developed automated WCAEE tool where it is stated that several engineering aspects have been considered to develop the proposed tool. In our development, we incorporated the updated version of the Web Content Accessibility Guideline (WCAG 2.2) which is the most appropriate web content accessibility evaluation guideline. Besides, as WCAG does not consider every end-user criterion, one of the earlier works examined how user and expert perspectives can aid in the accessibility evaluation process^10^. This indicates that taking these factors (user and expert perspectives) into account is a valuable resource for enhancing the accuracy of the accessibility investigation process. Furthermore, we consider the knowledge simplification concept for WCAG 2.2 and user and expert-suggested criteria simplification as it is significantly important to improve the understandability of each success criterion to incorporate it into the coding process. The proposed tool is developed based on the categorization of all the evaluation aspects into semantic and non-semantic approaches that incorporate both WCAG 2.2 and user and expert-suggested criteria through an algorithmic evaluation process. Finally, the algorithmic evaluation process performs the complexity analysis to formulate the evaluation report.

The developed WCAEE tool consists of four phases, as shown in Fig. 1, where the development process involves: (1) modeling the structural elements to facilitate the structuring of the guidelines and user and expert-suggested criteria; and (2) conducting a complexity analysis by loading the webpage and implementing three distinct complexity analysis algorithms. Besides, the implementation process involves: (3) formulating the report to provide overall evaluation statistics that include feedback and results related to each checkpoint of the implemented guidelines, as well as information on conformance level, impairment types, and direction for further improvement through four distinct window views; and (4) visualizing the report through various graphical analysis (graphs, charts, and other visual representations). The ensuing subsections provide a detailed description of the development and implementation process.

#### WCAEE tool development

The WCAEE tool development performs by integrating structural elements from both WCAG 2.2 and user and expert suggestions as it is crucial to determine the appropriate variables related to each web object corresponding to each success criteria and user and expert suggested criteria for performing the complexity analysis to determine the accessibility issues. All the structural elements are classified into Non-Textual elements (WCAG 2.2), Textual elements (WCAG 2.2), and additional elements (user and expert suggested criteria). After determining the structural elements or variable identification, an algorithmic evaluation was performed incorporating three distinct algorithms (Non-Text Complexity Analysis Algorithm, Text Complexity Analysis Algorithm, Additional Criteria Validation Algorithm) for performing the complexity analysis to determine the actual accessibility score and associated accessibility issues. The following subsections describe the development process in detail.

##### Structural elements: variable identification for complexity analysis

During the development process, we considered standard web content accessibility guidelines and user and expert assessments or requirements for determining structural elements as these aspects provide a detailed view of important variables, also known as webpage objects, which are regarded as prime assets for evaluating webpage accessibility. We evaluated the most recent version of the widely accepted web content accessibility guidelines (WCAG 2.2) and conducted a user and expert study to ascertain their requirements and assess the associated objects. In that case, it directed us to understand how web content accessibility guidelines and user and expert suggestions help to determine the structural elements to improve the accessibility issues or complexity.

The WCAG 2.2 assessment process emphasized the necessity of simplifying or modeling the guidelines because they are written in a natural language format and can be challenging to comprehend when attempting to develop evaluation tools or incorporate the guidelines into the evaluation process. As a result, we developed AccGuideLiner, an advanced engineering tool, to simplify all 87 success criteria. This tool provides a complete view of simplified guidelines for a variety of criteria, including beneficiary type, evaluation type/phase, attribute, component type, requirements, and conformance level. The AccGuideLiner tool is one of our works that has been developed to support our WCAEE tool development. The Tkinter GUI toolkit (https://docs.python.org/3/library/tkinter.html) was utilized in the development of AccGuideLiner within a Python environment. The details of the developed AccGuideLiner tool can be found in^39^. In guideline modeling, we referred objects to the specific webpage features that addressed particular success criteria; attributes indicated whether the variable was an HTML or CSS tag; the component type referred to textual (title, paragraph, etc.) and non-textual (header, footer, color, etc.) webpage features; requirements referred to information that was missing but could help to improve the issues that were detected; conformance level which referred to priority/importance of the success criteria; beneficiary type referred to the target audience or users connected with special requirements, and for whom that particular success criterion should be satisfied and evaluation type/phase referred to whether a specific success criterion can be evaluated automatically or requires manual evaluation. From guideline modeling, we found 28 objects or structural elements that relate to 51 of the 87 WCAG 2.2 success criteria that are inherently evaluable. Based on our knowledge, the remaining 36 success criteria need to be manually assessed. The identified 28 variables are classified into non-textual elements and textual elements which have been provided in the following sections. Besides, all the rules or directions about each addressed non-textual and textual element of web content accessibility guidelines (WCAG 2.2) are given in Table B.1 (Table 1; Appendix B).

**Non-textual elements**

**[Audio/Video]:** When compared to text alone, audio and video are thought to be valuable mediums for representing information. Therefore, it is advised that audio and video files be included in the webpage to better convey the content and increase webpage usability and accessibility. We assessed the presence of any audio and video material through ‘audio’/ ‘video’ elements with ‘controls’, and ‘src’ attributes.

**[Links]:** There are two types of hyperlinks (internal and external) that can be used to navigate webpages. Internal and external links are assessed in terms of their presence and assignment properly, as well as for any broken or unavailable conditions and appropriate length of observation. A maximum of 80 characters was taken into consideration when determining the link’s length. Additionally, we used 'a' and ‘link’ elements with the ‘href’ property to analyze hyperlinks as well as links that point to other CSS files based on their name, role, and value.

**[Display Orientation]:** Referenced width is used to evaluate display orientation; for example, width should be set as device width, and the default or starting scale should be 1. The availability or visibility of webpages across a variety of platforms, including laptops, desktops, tablets, mobile devices, etc., can be enhanced by properly indicating orientation assignments.

**[Input field]:** Input fields are the most common objects of webpages that require input based on their type. Therefore, a clearly stated purpose is essential to improve its usefulness to the people and make it understandable and accessible. All the input fields of the webpages are evaluated through ‘placeholder’ and ‘aria-label’ attributes as they indicate or instruct the actual purpose of the input field.

**[Buttons]:** According to accessibility, buttons need to be labeled in a way that is accessible to those with disabilities such as people with visual impairments who use assistive technologies for navigating webpages. To evaluate the accessibility of buttons, we evaluated through the ‘aria-label’ attribute as this attribute specifies button purpose and makes the navigation process easier for people.

**[Headings]:** Headings are crucial components for accurately representing and enhancing the information’s semantic quality. Inappropriate background and text color choices may make it challenging to correctly comprehend the information being displayed. Since red–black and red-green are the most challenging color combinations for individuals with special needs to navigate, we thus assessed the heading color in terms of these two-color combinations.

**[Header]:** The header is one of the webpage’s secondary core components, or characteristics, that is most crucial to accurately reflect the content. Selecting the wrong background and text color can make it difficult to accurately understand the information that is being shown. Thus, all the headers were assessed based on the way the background and text colors blended. Similar to headings, all of the headers were assessed using two-color combinations, including red–black and red-green.

**[Paragraph]:** A paragraph is made up of several sentences put together to give the user all the information in the site content. Therefore, clearly representing this text is important. In majority of the time inaccessible color makes this text representation difficult for a specific group of people such as color-blind people or people who use assistive technology for navigating the webpages. Thus, the background and text colors of the paragraphs were assessed in relation to two-color combinations, including red–black and red-green, which are reminiscent of the header.

**[Background Music]:** Background music is one of the responsible elements that contribute to accessibility issues. Therefore, it was advised by accessibility experts to avoid using any background music or audio to make web content accessible. We assessed background music using the HTML code’s “bgsound” element.

**[Keyboard access]:** For people with special needs, having keyboard access is one of the most important requirements for an efficient online navigating experience. Keyboard access functionalities (Tab key) were evaluated through ‘tabindex’ global attribute with default 0 and − 1 values for lang, title, link, div, header, ul, li, button, paragraph, heading, img, footer, and other attributes.

**[Keyboard Character Key Shortcut]:** People with special needs who use various assistive technologies to access webpages may encounter challenges if a webpage’s content has designated keyboard character shortcuts which is referred to as a hidden trap. The ‘accesskey’ global attribute was used to assess keyboard character shortcuts for each element, including buttons, paragraphs, headings, labels, headers, footers, li, ul, and other elements.

**[Search Field]:** The search field is used in the webpages for consistent identification and navigation. Sometimes, search fields are not properly defined, and are not understandable and navigable, thus we assessed search field understandability using the “placeholder” element and active status using the “action” attribute. Besides, we used the ‘role’ attribute to assess the functionality of the status message.

**[Label]:** A descriptive, meaningful, and appropriate label description plays a crucial role in improving its accessibility. Among several techniques, a properly specified label name might be most effective and can improve its accessibility; thus, we assessed label name specification using the ‘for’ property on the ‘label’ element.

**[Dropdown Menu]:** Dropdown menus come in a variety of forms, including clicking and mouse hovering. However, in some cases, the dropdown menu’s usefulness might be diminished because of the assigned elements include missing information that causes the menu to behave improperly. In order to make sure that no information is missing for any of its elements and attributes, we examined each element using the “label”, “select”, “option”, and “value” attribute.

**[Dialog box]:** The dialog box facilitates user involvement by providing instructions on how to resolve a particular problem, which is especially helpful when making decisions. In some cases, the dialog box returns null values due to missing information in the dialog box element. To confirm this, we assessed the ‘dialog’ element or tag considering its assigned information.

**[Form]:** Forms act as an important tool for collecting user information. Forms might be frequently malfunction because of a lack of instructions regarding expected input data. As a result, we assessed forms using two criteria: “label” to specify the type of data that should be entered, and “action” to indicate that the form is active and operating as intended. Additionally, the form’s status message was assessed using a “role” property, similar to the search field.

**[Status Message]:** Status message refers to the return message from the server while navigating the webpages. Sending or returning status messages from the server for every request represents the responsiveness of the server. Through the status message, the user is informed of the status of a particular action. In our system, we evaluate the status message of all the input and search fields to evaluate their accessibility status. We considered the “role” attribute while assessing the status message functionalities of any input fields or search fields to ascertain the functionalities they offered.

**[Error Message]:** If an error occurs, an error message must be displayed to explain the issue to inform the user of the error’s cause and suggest additional actions. To enable error generation, the defined error message needs to be suitable and accurately convey the command. To evaluate the error message accessibility, an error detection function, such as the true or false status of the defined "aria-invalid" attribute of the “input” element was considered to evaluate.

**[Error Suggestion]:** According to WCAG, if an input error is automatically identified and the possible correction suggestions are detectable, then the automatic suggestions are provided to the user. However, among several solutions, some most common and effective tags used in HTML code can be useful to evaluate error suggestion functionalities. Thus, to evaluate the recommended error suggestion, we considered the ‘span’ tag within the ‘input’ element.

**Textual elements**

**[Image]:** One of the essential components of web content that is regularly utilized to provide readers with descriptive information is an image. Images (Image, Gif, Animations, Logos, and decorative images) were evaluated through their alternative text whether it is assigned or not, as assistive technology users completely rely on it to comprehend the content of the images. We assessed this by validating its alternate text using the ‘image’ element with the ‘alt’ attribute.

**[Pre-recorded/Live Audio and Video]:** These days, webpages usually have live or pre-recorded audio and video content attached in order to facilitate sharing information with the user. Users can effectively and valuably use the represented information using audio and video content. Information received by audio or video is more advantageous than information received through text, particularly for individuals with disabilities. Thus, all audio/video content (pre-recorded or radio webcasts) was assessed based on its accurate caption and a clear explanation that aids in resolving accessibility concerns. The pre-recorded/live Audio/Video was evaluated through ‘audio’/ ‘video’ elements with ‘audio controls’, ‘video controls’, and ‘alt’ attributes.

**[Title]:** Webpage titles also known as meta titles or title tags. The title of the webpage reflects its purpose to the user. In HTML page, the title is defined by the title element. Thus, in the proposed system, the title was extracted using the ‘title’ element. As the title represents the purpose of a particular webpage, thus, we assessed its presence and to ascertain whether the title is accurate, descriptive, and appropriate, we evaluate whether it follows a coherent or meaningful sequence.

**[Words]:** Words are the normal text that is used to represent the content or information of a webpage. The content or information presented should be clear and understandable in order to increase accessibility. Here, all the words of title and body content were evaluated in terms of their meaningfulness and word spacing ratio where we considered 0.16*12pt as proper spacing between two words.

**[Paragraph]:** A paragraph is the body of a webpage which is also known as a descriptive explanation of the information. As this is one of the prime content to understand the purpose of a webpage, it should be meaningful, simple, precise, and contain useful but sufficient information that will help to understand the content. To evaluate their accessibility, the entire paragraph of a webpage has been extracted with the help of the 'p' element. Similar to the title, the body content was assessed based on the meaning sequence observation, which aided in determining its efficacy for the intended audience.

**[Webpage Text]:** The text on the webpage refers to the title and body of the text that is assessed using a number of semantic methodologies, including reading level, repetitions/redundancies, unusual words, acronyms, and mispronounced terms. The ability of the user to read the text without difficulty is referred to their reading level, particularly for individuals with disabilities. Sustaining reading levels is crucial for enhancing web content accessibility. The ability to comprehend words or sentences without trouble is known as pronunciation. Sentences and meaningful words are essential for improving pronunciation ability. Complex and ambiguous words ought to be avoided in the text content of webpages. The shortened version of words or phrases is called an acronym. An example of an acronym is IT, which stands for information technology. While abbreviations might be useful in some contexts, using such condensed versions is inappropriate for accessibility because persons with impairments often struggle with these shorter forms of words or phrases. To grasp the meaning of an abbreviation, it is vital to supply a broad and extended form when it is unavoidable.

**[Buttons]:** The button is one of the advanced user interface components that help users interact with the system with a single tap. However, improper information such as non-clear functionalities makes the navigation process inaccessible. Therefore, we evaluated buttons in terms of their defined purpose through the ‘button’ tag using the ‘title’ attribute.

**[Links]:** Links are additional resources on a webpage that are intended to expand or improve the user’s understanding of the content on the page. A webpage may include a number of links, both internal and external, to expand its content. All the internal and external links were assessed based on their stated purposes since these serve to illustrate the links’ usefulness. Using the “title” attribute on the “href” element, we assessed the purpose of the link.

**[Headings and Labels]:** The headings and labels are the secondary vital aspects or qualities of the webpage that are most crucial for accurately expressing the information. A descriptive, meaningful, and appropriate description of headers and labels is suggested to enhance web accessibility and navigation process. We assessed headings and labels according to their stated purposes. The purpose was evaluated through their text component.

**[Language]:** Properly defined language is a basic requirement of any webpages. In some cases, developers prefer to define several sections or content through different languages hindering the consistency of the presented information. According to the accessibility perspective, using multiple languages on one webpage reduces the content’s readability and consistency. Keeping a single language for all sections and paragraphs of the content is another crucial feature of accessibility that needs to be upheld. Thus, it is not advised to use different languages in content as this decreases webpage accessibility and makes a webpage less programmatically determinable. To ascertain whether the content is written in a single language or a blend of several languages, we assessed it using lang detector.

**[Checkbox]:** In webpage, a square box with the ability to be checked or marked for any active operation is known as checkbox. In many cases, missing information raises challenges in deciphering the required input data, similar to a dropdown menu. To determine if any missing input data was discovered, the input data was assessed using “input” and “checkbox” components with “value” attributes.

**[Combo boxes]:** A unique feature of webpage is the combo box, which lets users select an item from a vast list of possibilities to make it easier for them to find what they’re looking for. Unfortunately, developers often forget to specify the correct name, which makes it challenging for those who use assistive technology. Accessibility problems are exacerbated when information in the presented list is missing. Thus, we used the "input," "select," and “option” elements with their “name” and “value” properties to assess combo boxes based on their supplied name and item list.

**Additional elements**

A number of studies have found that following web content accessibility guidelines alone is insufficient to address all problems pertaining to individuals with special needs^40,41^. These studies have emphasized alternative approaches along with web content accessibility guidelines to present a more comprehensive picture of online accessibility. As a result, we consulted/interviewed ten users and three specialists/experts to get their insightful opinions on how to enhance the functionality of the web accessibility assessment tool. We especially requested experts for their opinions on any extra factors that could be useful to add to the evaluation process but weren’t covered in WCAG 2.2.

For selecting the potential users, an open invitation was circulated on the university’s official Facebook page with the details about our aim to conduct the interview. We selected the potential user according to some criteria such as -being active in the online platform, and -having sufficient knowledge about webpage browsing. According to the selected criteria, 8 users were selected including 6 students (M.Sc. students are aged between 24–28) and 2 end-users (2 persons are service holders aged between 35–42) who frequently access webpages for their daily activities including studies, official work, and personal activities. Additionally, through our university stuff connection, we found 2 older adults aged between 63–71 who were interested in providing their feedback as at this age they are most likely to depend on online platforms for arranging their daily activities like shopping, banking, etc. Thus, in total, we interviewed 10 users to get their responses or feedback. Two questions were asked during the interview and requested to provide their valuable opinion. The interview was taken via Zoom meeting, and it took 30–35 min on average for each. The questions that were asked are as follows.

- Q-1: What kind of features would you expect that could be helpful to navigate the webpage from your perspective?
- Q-2: Why do you prefer these features? How could it help in navigating the webpage?

All the participants mentioned several aspects that are encountered frequently during webpage accessing and introduced accessibility issues. However, many of their suggested criteria are related to WCAG, thus, those were excluded, and considered only 5 criteria that are unique, and web content accessibility guidelines, including WCAG, do not address these aspects. The determined 5 criteria are manual ‘text size’ and ‘font adjustment’ availability, manual ‘color adjustment’ availability, ‘necessity of user information’, and ‘availability of textual and image CAPTCHA’. However, the questionnaire used in this work concerning these aspects helps us understand the user’s perspective and obtain their particular requirement regarding every single aspect. Also, understanding user requirements might be helpful to improve the overall accessibility evaluation process.

Furthermore, for selecting the potential experts, first, we have chosen some experts from our professional network who are working in the field of digital accessibility including ‘web accessibility’ and ‘web evaluation’. Also, we searched for professionals in ResearchGate directories according to expertise in the field of ‘web accessibility’, ‘digital accessibility’, ‘universal inclusion’, and ‘inclusion of digital platform’. Besides the field of expertise, we also considered the number of years of research experience in the particular field (expected at least 5 years of experience), and the quality of scientific publication. Primarily we found 10 experts/professionals from our network and ResearchGate profile from different countries who are working in different universities and research centers as academics/researchers/senior researchers. We invited them through a professional email where we provided the project description and the objective of conducting the interview. However, we got a positive response from four professionals although one professional couldn’t attend the interview due to his/her personal issue.

The selected three experts were from Nottingham Trent University, UK; University of Maryland, USA; and Jahangirnagar University, Bangladesh. Two experts have more than 20 years of experience in the domain of accessibility of digital platforms and one expert has more than 5 years of experience in this field. They are aware of all versions of WCAG and have sufficient knowledge and experience to work with WCAG. To conduct this interview, we selected three questions that we asked during the interview and requested to provide their insightful suggestions. The interview was taken via Zoom meeting and the overall interview took 45–56 min for each person. The selected questions are as follows:

- **Q-1:** What kind of arbitrary information do you think is useful to improve the performance of the automated accessibility evaluation results that are not mentioned in WCAG?
- **Q-2:** What kind of non-arbitrary information has an impact on improving the effectiveness of the automated accessibility evaluation results that are not included in WCAG?
- **Q-3:** Why do you suggest these attributes? How it facilitates to improve the performance, please explain.

Three experts present twelve (12) criteria with a brief explanation of the importance of these criteria based on their knowledge and experience. They recommended that these criteria should be considered when developing our proposed tool in conjunction with the most recent version of the web content accessibility guidelines (WCAG 2.2).

After assessing users’ and experts’ suggestions, we identified 10 additional variables for 17 additional criteria (5 from user and 12 from expert) that are crucial to consider in the proposed web accessibility evaluation process to address the seventeen identified criteria. The additional 10 variables are described in the following. Besides, all the rules or directions about each addressed additional elements for web content accessibility evaluation are given in Table B.1 (Table 1; Appendix B).

**[Loading time]:** The average amount of time it takes for a webpage to load when a user searches or browses through their web address in the search panel is known as the webpage loading time. Webpage loading time acts as a crucial factor in webpage accessibility because users will become dissatisfied and webpage interactivity may be excluded if a page has problems and takes longer than usual to load. Thus, we evaluated the webpage loading time considering the standard or average loading time of 0.3 s.

**[Paragraph length]:** A lengthy paragraph introduces difficulties in understanding web content for people with cognitive disability. To make the web content accessible to every group of people, it is recommended to limit its length to make it compressive to the user. We evaluated the paragraph length according to the standard ratio of 1500 words maximum.

**[Hyperlink ratio]:** Hyperlinks are essential components of the webpage that give the user access to more information. However, individuals with special needs find it challenging to navigate webpages with an excessive number of hyperlinks in the text. Experts in accessibility estimated that no more than fifty hyperlinks are necessary to accurately convey the content and give the user all the information they need.

**[Default Language]:** Webpage default language was evaluated considering the English language of the webpage. As English is the global language, setting it as the default language on a webpage can increase its accessibility for the general public. We evaluated the language of the webpage through the ‘lang’ attribute to determine whether the webpage has an English version.

**[Webpage length]:** It refers to the duration of a webpage including both its content and display sizes which influence the navigation time. Similar to webpage loading time, webpage length plays a critical role in enhancing accessibility. People with disabilities may find it very difficult to use lengthy webpages due to mobility issues or cognitive difficulties that prevent them from viewing content for extended periods of time. Thus, sufficient information should be appropriately arranged within a set of number of pages. According to the accessibility perspective, we evaluated the webpage length with a limit of 14 KB in mind.

**[Server Status/Availability]:** The purpose of webpage availability, also known as webpage uptime, is to ensure that users may view or navigate the page whenever they choose. In the event that a webpage is unavailable, its effectiveness could be diminished, and users could be less inclined to return often. Consequently, keeping a webpage up to date is essential to improving accessibility. The severe condition/availability was assessed in terms of being down, deactivated, or active. A key contributing factor to a webpage’s accessibility reduction is that most users become disinterested in returning to it when the server is unavailable.

**[User information]:** In general, user information relates to whether accessing a webpage involves logging in or registering. Certain webpages may ask for personal information from their users, including their location, email address, password, username, interests, etc. These make the webpage inaccessible as users might not consent to sharing their information and users with disabilities might misunderstand what these criteria actually entail. In that case, users might not want to continue this process to browse the page. Thus, we used login functionality to assess whether the webpage needed any login or registration information.

**[CAPTCH]:** Recently, CAPTCHA-enabled webpages have become increasingly popular, either for security reasons or to better understand user behavior. While some users find image-based CAPTCHA to be more helpful, others prefer text-based CAPTCHA. However, it is one of the hardest tasks for people with special needs which reduces webpage accessibility. To evaluate the accessibility of webpages in terms of the presence of CAPTCHA, we used the ‘div’ element with the ‘id’ attribute to determine whether CAPTCHA was present or being used.

**[Multiple languages]:** Language is a unifying element of webpage that allows the community to access it from anywhere in the world. For a webpage to be user-accessible, it must be available in several languages and include a selection choice. To attain this goal, using “nav” and “ul” elements with “li” and "a" attributes, the multiple language option was assessed to see if the webpage specified multiple languages or not to make the webpage available to users of various languages.

**[Image ratio]:** As previously mentioned, image is an essential component of webpage. However, it may raise accessibility concerns, if the proper placement ratio is not maintained. Overuse of graphics or images in webpage content may make a webpage less accessible to users with cognitive impairments. Therefore, according to accessibility experts, a limit of 10 images can be a reasonable decision to preserve its accessibility from an accessibility standpoint.

**[Manual font adjustment option]:** Enabling manual adjustments to text size or font size is a primary cause of decreased accessibility. As font type varies from user to user, thus the webpage was evaluated to see if it had a stated font adjustment option that allowed users to manually change the font to suit their preferences.

**[Manual color adjustment option]:** Similar to font type, the type of color also varies from user to user. So, we evaluated whether the page allowed the color adjustment option to be adjusted manually in terms of the user interest.

**[Text Font family]:** An appropriate font family should be used as the default font family for webpage content in order to make the content accessible to people with all types of disabilities. Among several font families, only a few of them are considered as accessible to make the content accessible to every group of users. We evaluated web content text and heading text in terms of six specific font families such as Tahoma, Calibri, Helvetica, Arial, Verdana, and Times New Roman.

**[Text Font size]:** To ensure content accessibility, the default font size of webpage content needs to be appropriate for all categories of individuals with disabilities. The accessibility expert stated that text font sizes should be 16 px (12 pt) or larger, thus we assessed the textual content’s (header and body text) accessible font size by comparing it to the (≥ 16 px) ratio.

**[Text pattern]:** Text pattern refers to different styles of text representation such as italic, bold, etc. Unsuitable textual conventions may make the content challenging to read. For example, the “italic” text pattern can be perplexing to certain people. As a result, using suitable text patterns may make the content easier to understand and more accessible. The text pattern was evaluated considering ‘bold’, ‘strong’, ‘Italic’, ‘emphasized’, ‘marked’, ‘subscript’ and ‘superscript’ formats through ‘b’, ‘strong’, ‘i’, ‘em’, ‘mark’, ‘sub’ and ‘sup’ attributes.

**[Content type]:** Content type refers to web content that should have a combination of textual content, image content, and video or audio content. To represent web content effectively to people with special needs, following the appropriate content type is essential to improve its accessibility to every group of people.

**[Number of audio/video content]:** Similar to the image content, an excessive amount of audio and video content might reduce the accessibility of the web content. Therefore, in the expert’s opinion, a maximum of 2 audio or videos is the optimal number of audio/video content that can improve the accessibility of the web content.

##### Accessibility evaluation: complexity analysis of structural elements

**Fig. 2:**
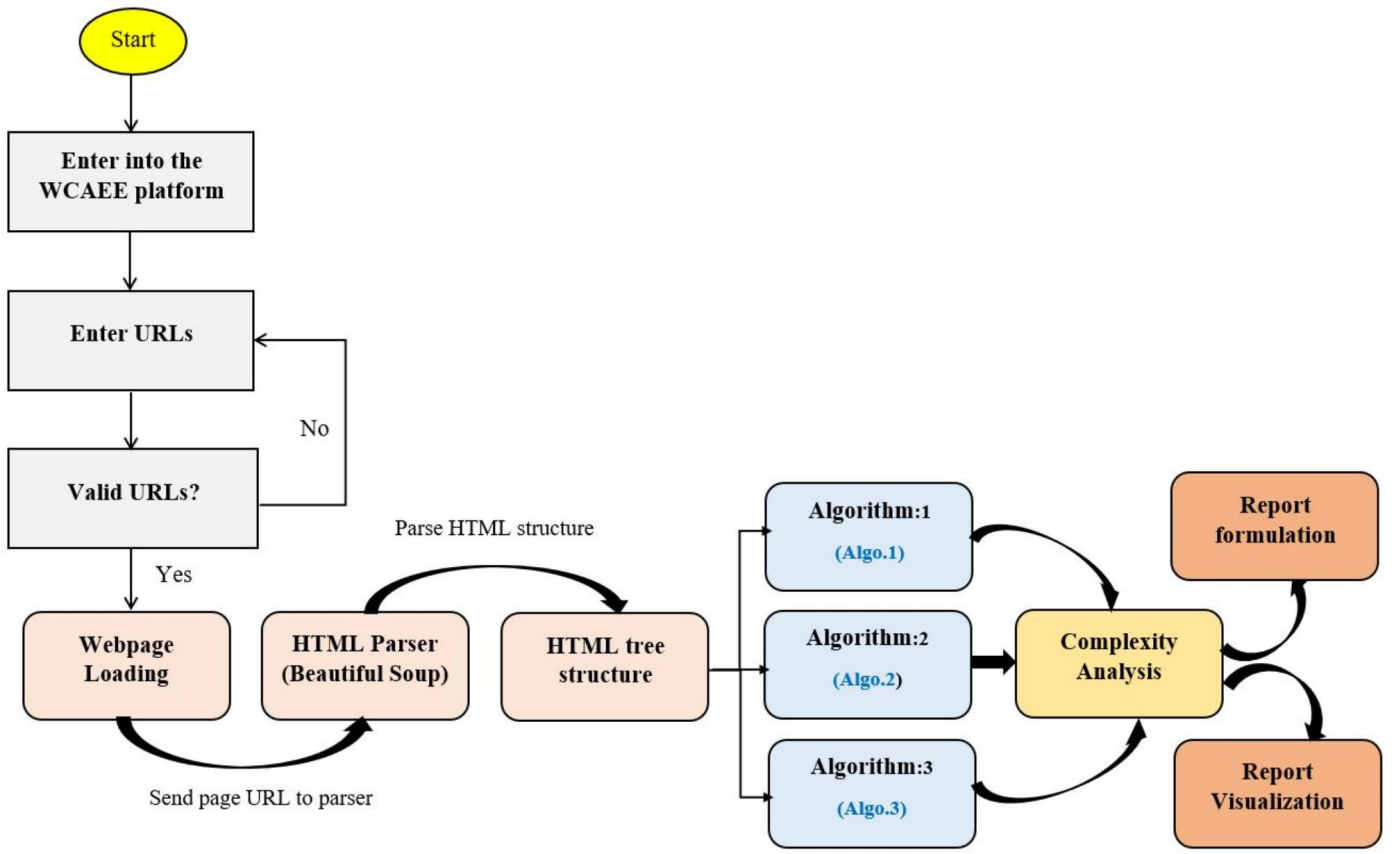
The work-flow diagram of the algorithmic evaluation process.

As we illustrated in Fig. 1, after finalizing the structural elements, algorithmic evaluation was carried out to analyze the complexity of particular web elements considering the accessibility perspective. The developed tool technically performs the accessibility evaluation based on three different algorithms: (Algo.1) Non-Text Complexity Analysis Algorithm (see section "Non-textual elements"); (Algo.2) Text Complexity Analysis Algorithm (see section "Textual elements"); and (Algo.3) Additional Criteria Validation Algorithm (see section "Additional elements"). The Text Complexity Analysis Algorithm assessed the complexity of the webpage’s textual components, while the Non-Text Complexity Analysis Algorithm assessed the difficulty of the non-textual elements of the tested webpage. Besides, the Additional Criteria Validation Algorithm highlights the complexity of the interactive components of the tested page. Figure 2 illustrates the workflow of the algorithmic evaluation process.

Figure 2 illustrates that to evaluates the accessibility of a particular webpage; the webpage’s URL is first validated in the WCAEE platform to determine its accessibility. Once the URL has been validated, it is sent to an HTML parser (in this case, Beautiful Soup) to parse the HTML code and convert it into the HTML Document Object Model (DOM) structure. The full HTML source code is represented by the DOM structure as a tree structure view, which may be utilized to navigate the HTML elements in accordance with the required HTML tags. To assess the complexity of the corresponding elements that refer to various online objects, three distinct algorithms have been implemented. We generated the evaluation report based on the results of the complexity analysis using three different algorithms, and we used graphical representation (a number of data visualization techniques) to make the report effective, interactive, and helpful to the end user. For implementing the developed WCAEE model, we considered sublime text editor as a development framework and Python programming language to write the script to implement the proposed algorithms. We took into consideration the Windows 10 version running on the eighth-generation Intel Core i7 processor. We utilized the Tkinter package to design user interfaces and numerous window views, as it is the most often used Python GUI library that enables the efficient creation of GUI programs. Additionally, an object-oriented interface such as the Tk GUI toolkit enables the integration of several objects into the user interface, which aids in creating and representing an interactive user interface for the user. We used a number of Python libraries, which are mentioned in (Appendix A), to traverse the tested webpage, parse the HTML code, extract the information such as elements and attributes, and carry out the full evaluation.

Three separate complexity analysis algorithms have been implemented to develop the WCAEE tool and carry out the overall evaluation procedure. The first algorithm is called the Non-Text Complexity Analysis Algorithm, and it evaluates the accessibility concerns of the webpage by considering its non-text components. The Non-Text Complexity analysis algorithm is able to assess 19 web objects in total, including their functionalities and other aspects (see section "Non-text-complexity analysis algorithms"). Similar to the first algorithm, the second one is called the Tex-Complexity Analysis Algorithm, and it analyzes all of the webpage’s text components to determine how complicated or problematic they are from an accessibility standpoint. The Text-Complexity Analysis Algorithm is able to assess a total of 12 web objects (see section "Text-complexity analysis algorithms") considering the textual components. After implementing these two algorithms, the additional criteria assessment algorithm has been used to analyze additional criteria that were received from user and expert assessment. It considers 17 web objects (refer to section "Additional criteria validation algorithm") to assess their accessibility perspective. The following sections provide a detailed explanation of our developed algorithms along with their evaluation strategies.

**Non-text-complexity analysis algorithms**

With particular attention to the elements and attributes of HTML objects, the Non-Text-Complexity analysis method navigates all web objects through HTML tags as it recursively explores the HTML tree view of the tested or provided webpage. The accessibility issues or complexity of a webpage certainly and mostly depends on or is related to the different elements coded into the structured document. Therefore, it is obvious that calculating the accessibility of the tested webpages and improving the quality of the evaluation process can be achieved by counting the occurrence or quantity of each element, evaluating element attributes, structural objects, and missing values of the presented elements on the document. As a result, we took into account these auxiliary techniques for the development strategies throughout the assessment process to assess the arbitrary data of the tested webpages to assess their accessibility requirements.

**Table 1:**
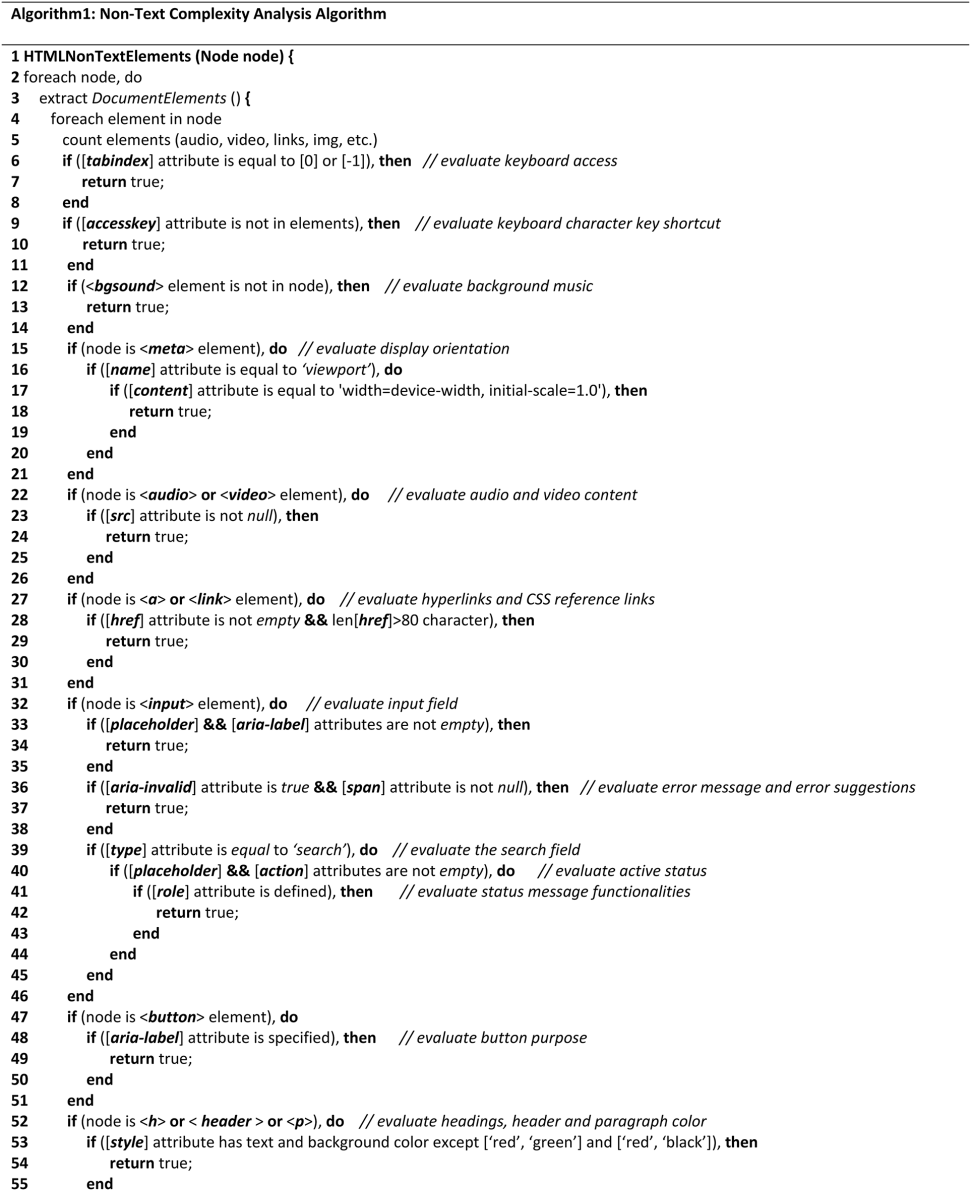

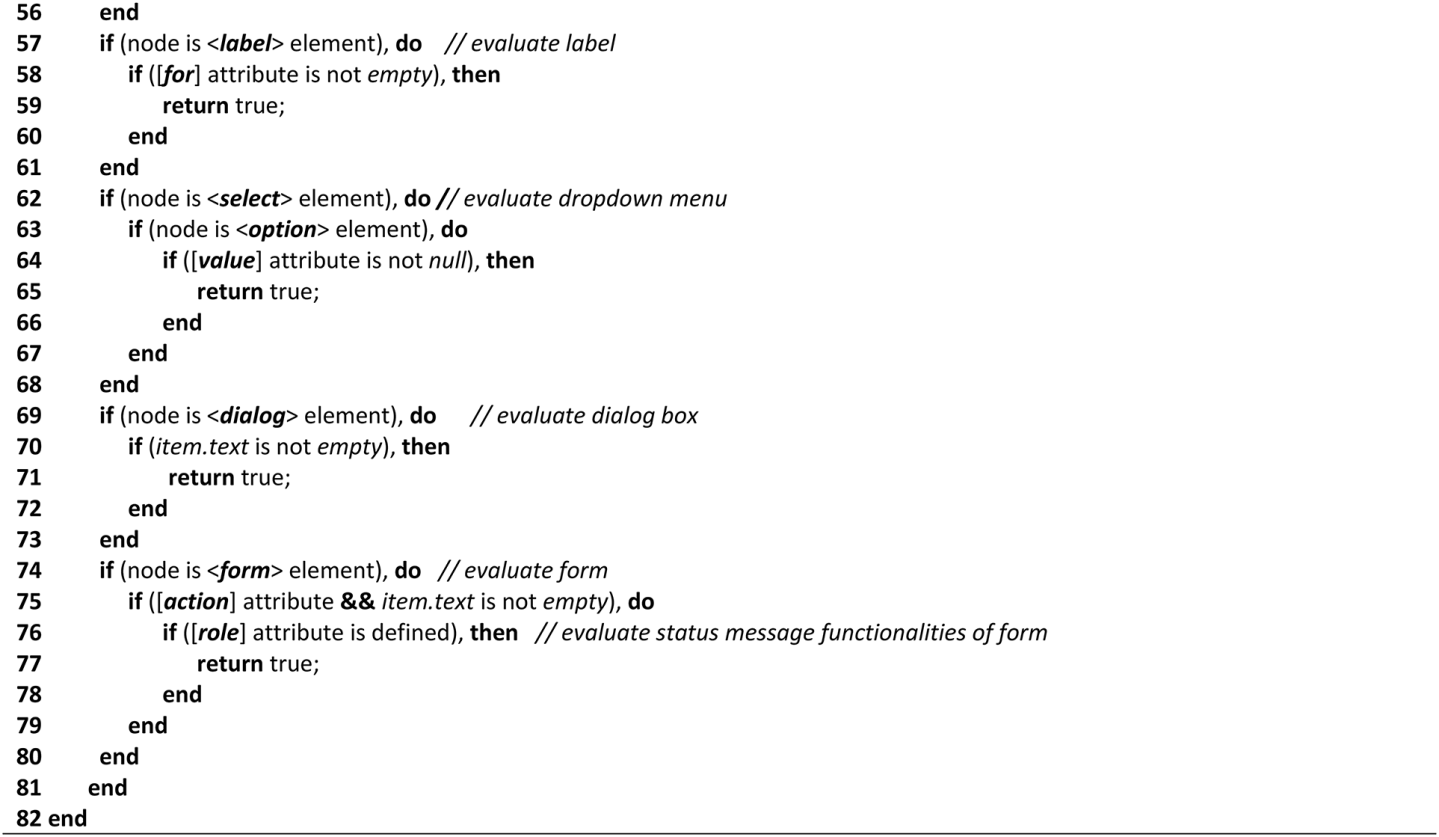
Shows the complexity analysis algorithm of Non-Text components.

As demonstrated in the (Algo.1) Non-Text Complexity Analysis Algorithm shown in Table 1, it conducts the evaluation process by executing multiple auxiliary methods to assess the objects and determine the accessibility score of the page. As the algorithm shows, in lines (2–4), the algorithm accesses each node in the HTML tree view to navigate the elements and attributes of each node. In line (5), it counts all the objects of the webpage as the counted statistic is used frequently in the evaluation process. In lines (6–8), it evaluates the keyboard access considering 0 and 1 values for navigation order, and also the keyboard character key shortcut was evaluated, in lines (9–11). The background sound or any background audio file was evaluated through the ‘bgsound’ element in the node, in lines (12–14). For evaluating the display orientation, it considers the device’s default width and 1.0 as an initial scale to identify as proper display orientation, in lines (15–21). Furthermore, it assesses the audio and video files in lines (22–26) based on their source attribute to ascertain their presence, as improper source files cause significant accessibility concerns. In lines (27–31), Hyperlinks and CSS reference links were evaluated in terms of their missing resources and length as longer than 80 characters might cause accessibility issues. The defined purposes of all input fields, including error message boxes and search fields, were assessed in lines (32–46). The normal input field was assessed based on its ‘aria-label’ and ‘placeholder’ attributes; error message boxes were assessed based on their ‘aria-invalid’ and ‘span’ attributes to determine the error message and error suggestion information; and the search field was assessed based on its placeholder, action, and role attributes to determine its defined purpose and executable functionalities. Also, lines (47–51) analyzed the buttons’ purpose using the ‘aria-label’ attribute. Besides, in lines (52–56), the color or headings, header, and paragraph were evaluated in terms of red-green and red–black color combinations as these are the most inaccessible colors according to the accessibility experts. To ascertain if the label has a clear name or purpose, in line (57–61), label name attribution was assessed. Lines (62–68) were used to evaluate Dropdown menu in terms of their assignment of missing values. The dialog box was also evaluated in lines (69–73), considering their missing value or information during the assignment. Lastly, all the form of the tested webpage was evaluated in lines (74–80) considering their text information, active status, and execution functionalities. Finally, the process of exploring the HTML tree view is stopped in lines (81–82).

**Text-complexity analysis algorithms**

**Table 2:**
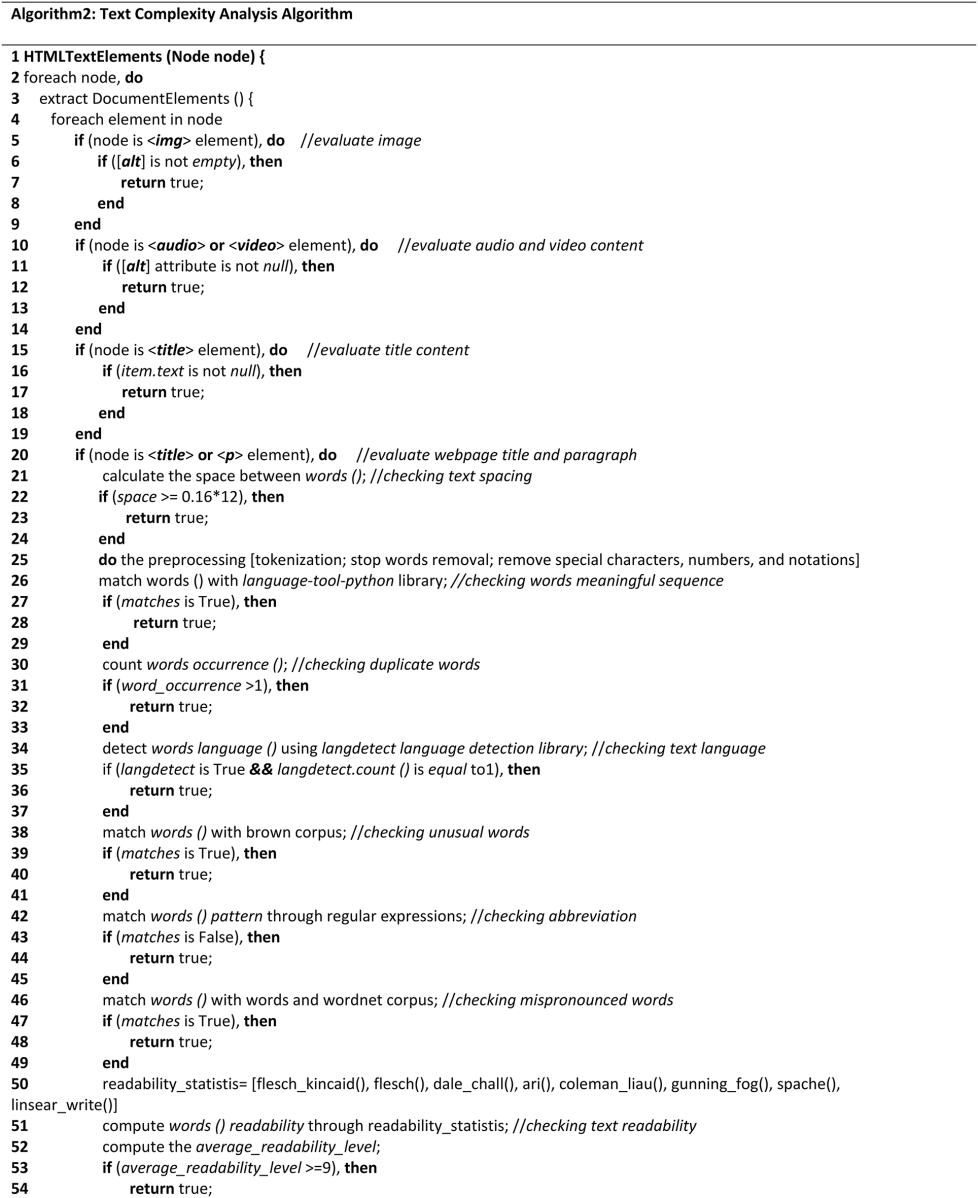

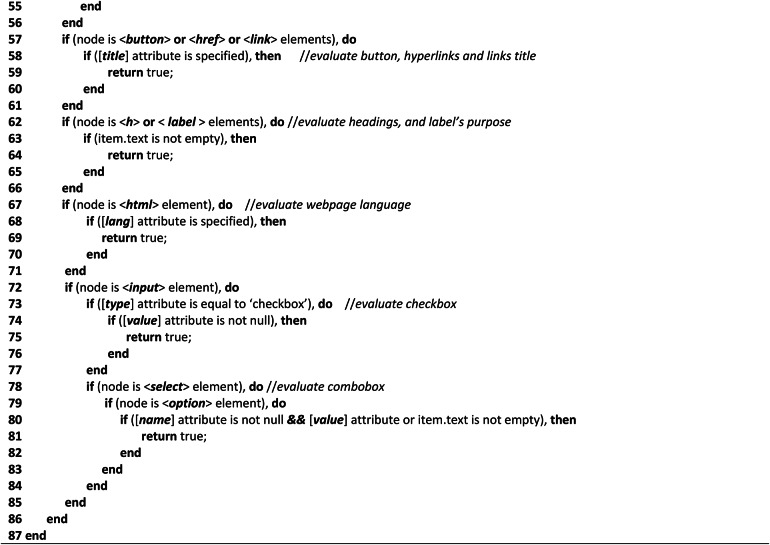
Shows the complexity analysis algorithm of Text components.

The Text-Complexity analysis algorithm, like Algo.1, traverses the HTML tree view of the tested webpage recursively to navigate all the elements and attributes of HTML objects. It assesses every textual element that is described in section "Textual elements". The Text-Complexity Algorithm as shown in Table 2 recursively identifies all the elements and their associated attributes (depending on the requirements) according to the selected variables chosen for textual components and performs the evaluation process, as the aim of this algorithm is to evaluate only the textual objects of the tested webpages. The Text Complexity Analysis Algorithm (Algo.2) (Table B.3 (Table 3; Appendix B)) comprises multiple auxiliary techniques and a number of Natural Language Processing (NLP) techniques for the evaluation process of non-arbitrary information (such as textual information) that assess multiple web objects to determine the page’s accessibility score. We considered different NLP techniques as it is considered one of the best methods for evaluating web material^11^. As the algorithm shows, in lines (2–4), it traverses the HTML tree view and accesses all the nodes to identify the elements and attributes of the tree view. In lines (5–9), it accesses all the image objects of the tested webpage using the img element and evaluates them in terms of their alternative text property. In lines (10–14), similar to the image alternative text property, the audio and video content are also evaluated considering a similar perspective. Lines (15–19) test the title of the webpage in terms of how accurately it describes and aids the user in understanding the webpage’s goal. In lines (20–56), it retrieves the title and each paragraph of the webpage and assesses the textual content or text information considering several aspects. Accordingly, in lines (21–24), the spacing within the text or between words was assessed using the 0.16*12 ratio, where 12 is the standard font size for all web content and 0.16 is a constant that indicates the amount of space that should be 0.16 times greater than the font size. We used ‘tokenization’ and stop word removal techniques from the nltk platform to preprocess the text in lines (25–29). To make the text cleaner, we also excluded special characters, digits, and annotations. After that, we checked the meaningful sequence of the words by applying the ‘language tool-python’ library. We count the word occurrences in lines (30–33) to identify any duplicate words because they can reduce the accessibility of the text. Additionally, we used the Python language identification library called ‘langdetect’ to identify the text language in lines (34–37), and we counted the identified language objects to determine the presence of multiple languages. Additionally, we used the ‘brown’ corpus in lines (38–41) to detect unique words; in lines (42–45), we considered regular expressions for abbreviation recognition; and in lines (46–49), we used the ‘words’ and ‘wordnet’ corpus from the nltk platform to identify mispronounced words. Additionally, to compute the readability of the text, in lines (50–55), we applied 8 readability observation statistics and considered their average score to determine the readability of the evaluated webpage text. We assess the assigned title property of the button, hyperlinks, and CSS reference links in lines (57–61) to ascertain whether they have appropriate titles by referring to their precise purpose. We assessed the function of headings and labels in lines (62–66) based on their textual characteristics. The webpage language was evaluated in lines (67–71) using their ‘lang’ property. All of the comboboxes and checkboxes were assessed on lines (72–85) based on the presence of their name and value attributes. Lines (86–87) are the closing of the nodes and elements of the nodes.

**Additional criteria validation algorithm**

The additional criteria validation algorithm performs the complexity analysis of interactive elements of the tested webpage through two important methods and a set of auxiliary methods. Referring to (Algo.3) as shown in Table 3, the first method ‘calculate (node)’ is a recursive method that performs arbitrary object evaluation. In lines (2–13), webpage loading time, length, and server active status are evaluated in terms of a specified ratio. The process of calculating the webpage loading time is demonstrated in lines (2–5), webpage length is demonstrated in lines (6–9), and the server’s active status is checked in lines (10–13). Similarly, the second method ‘count (node)’ counts page textual words, hyperlinks, images, audio, and videos as shown by lines (15–30). Once the elements have been counted, it is evaluated according to the determined ratio that has been designated as an accessible ratio. By iteratively going through each node in the HTML tree, this approach counts the number of page elements. From the auxiliary methods, we evaluated the webpage’s default language as English in lines (35–39). Using the ‘level’ and ‘div’ elements in lines (40–49), the requirements for user data and CAPTCHA were also assessed. Additionally, the ‘nav’ element was used to evaluate the multiple language options to see if the webpage has specified multiple languages to provide multilingual access in lines (50–57). Additionally, the font size and family were assessed on lines (58–67). Five font sizes and six widely recognized and accessible font families have been taken into account while assessing the text on the webpage. For text patterns, such as whether the text has any special text pattern that makes the content difficult for the user to understand, seven distinct patterns have been taken into consideration in lines (68–72). Finally, the content types such as the three major content types should be considered as mandatory types for any web content evaluated in lines (73–75). Lines (76–77) are the endings of the HTML tree node traversing.

**Table 3:**
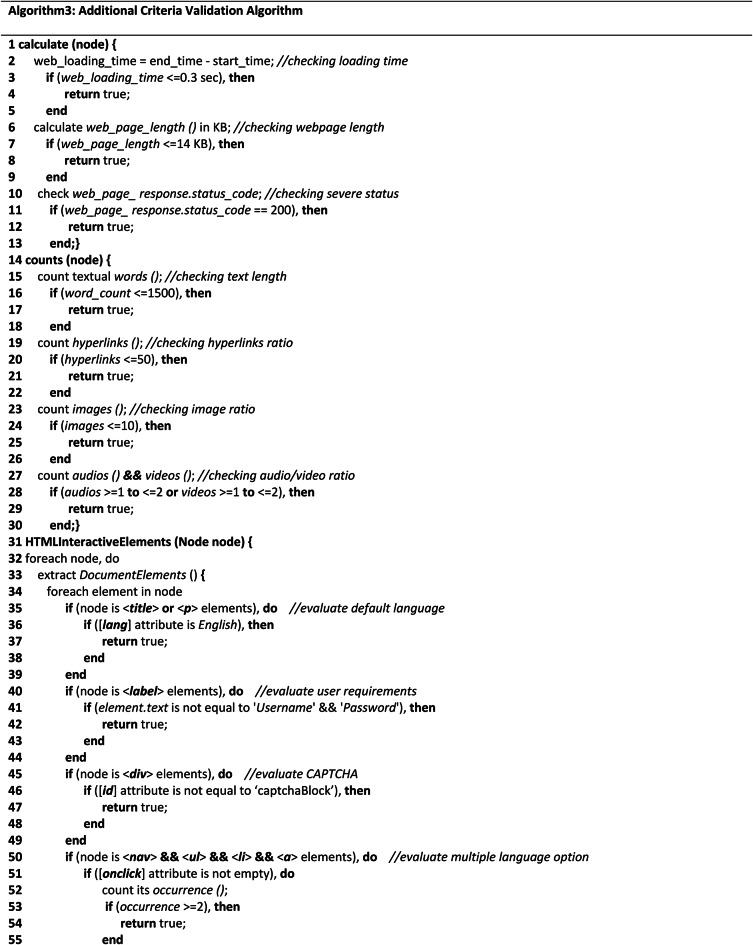

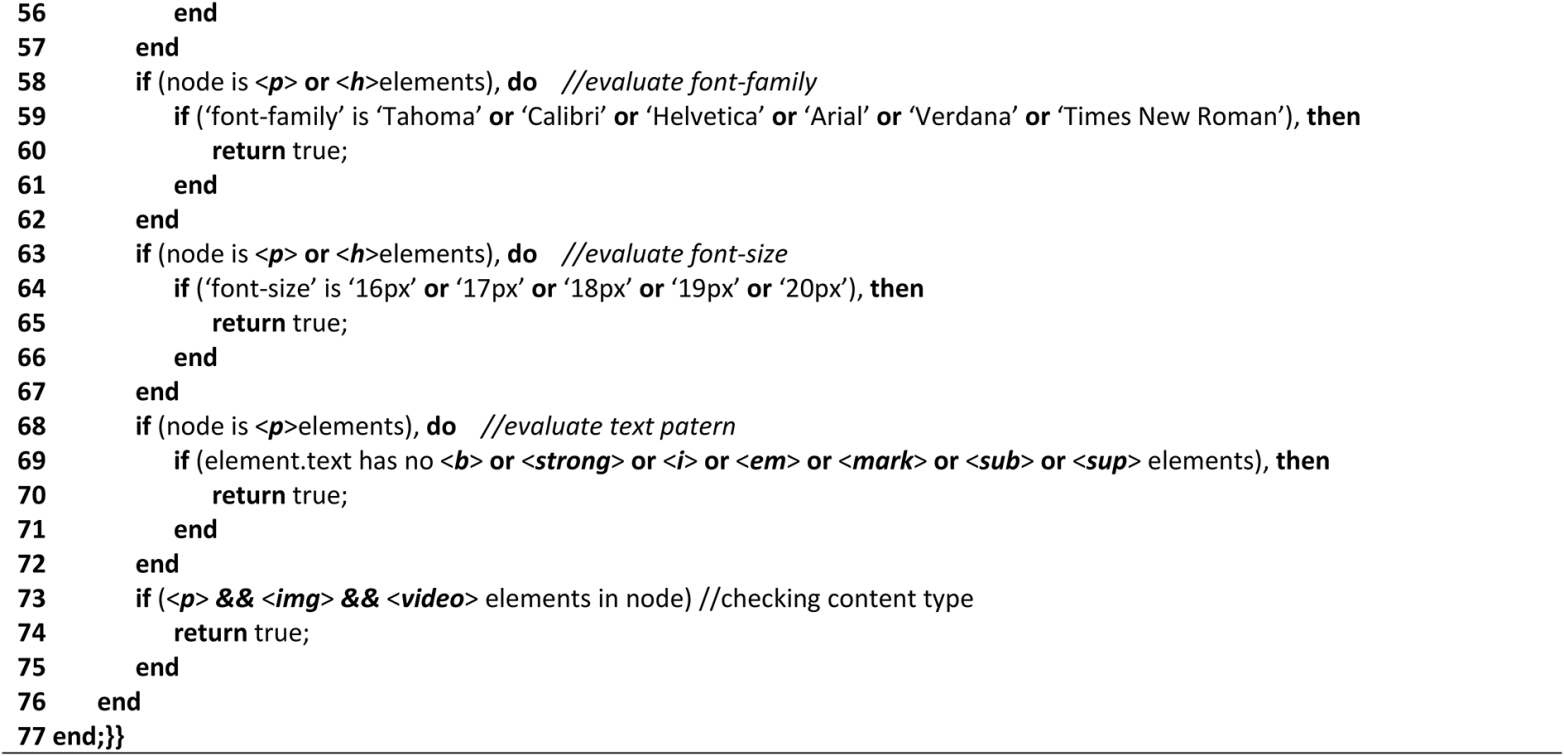
Shows the complexity analysis algorithm of additional components.

#### WCAEE tool implementation

**Fig. 3:**
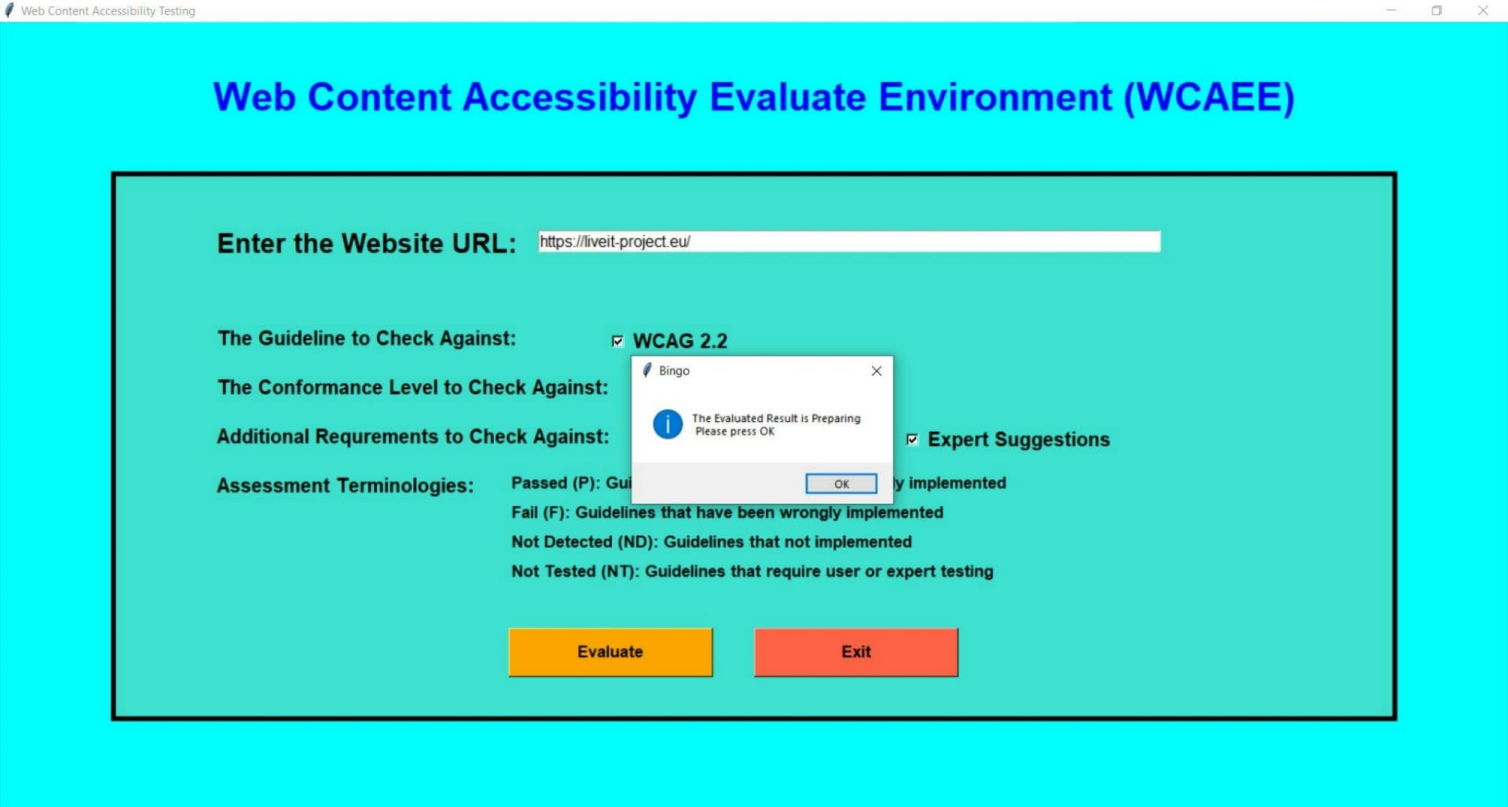
User interface of the WCAEE tool (URL validation).

This section aims to present the outcome of each complexity analysis algorithm for non-textual, textual, and interactive/additional elements. In order to implement the developed tool, the user’s initial step while using the WCAEE tool to assess a webpage’s accessibility is to type or paste the webpage’s URL into the URL validation screen. After that, the user must select/press the relevant button (Evaluate in orange) to proceed with the assessment procedure, as indicated in Fig. 3. The user must click “ok” to validate the warning after selecting the Evaluation button on the validation interface. Afterward, the URL of the page will be forwarded to the HTML parser, which translates the HTML source code into a tree structure including all of the element and attribute data. The three complexity analysis algorithms will then analyze each of the page’s objects or characteristics and generate a report with details about the accessibility concerns and complexity of the tested page.

##### Evaluation report formulation

**Fig. 4:**
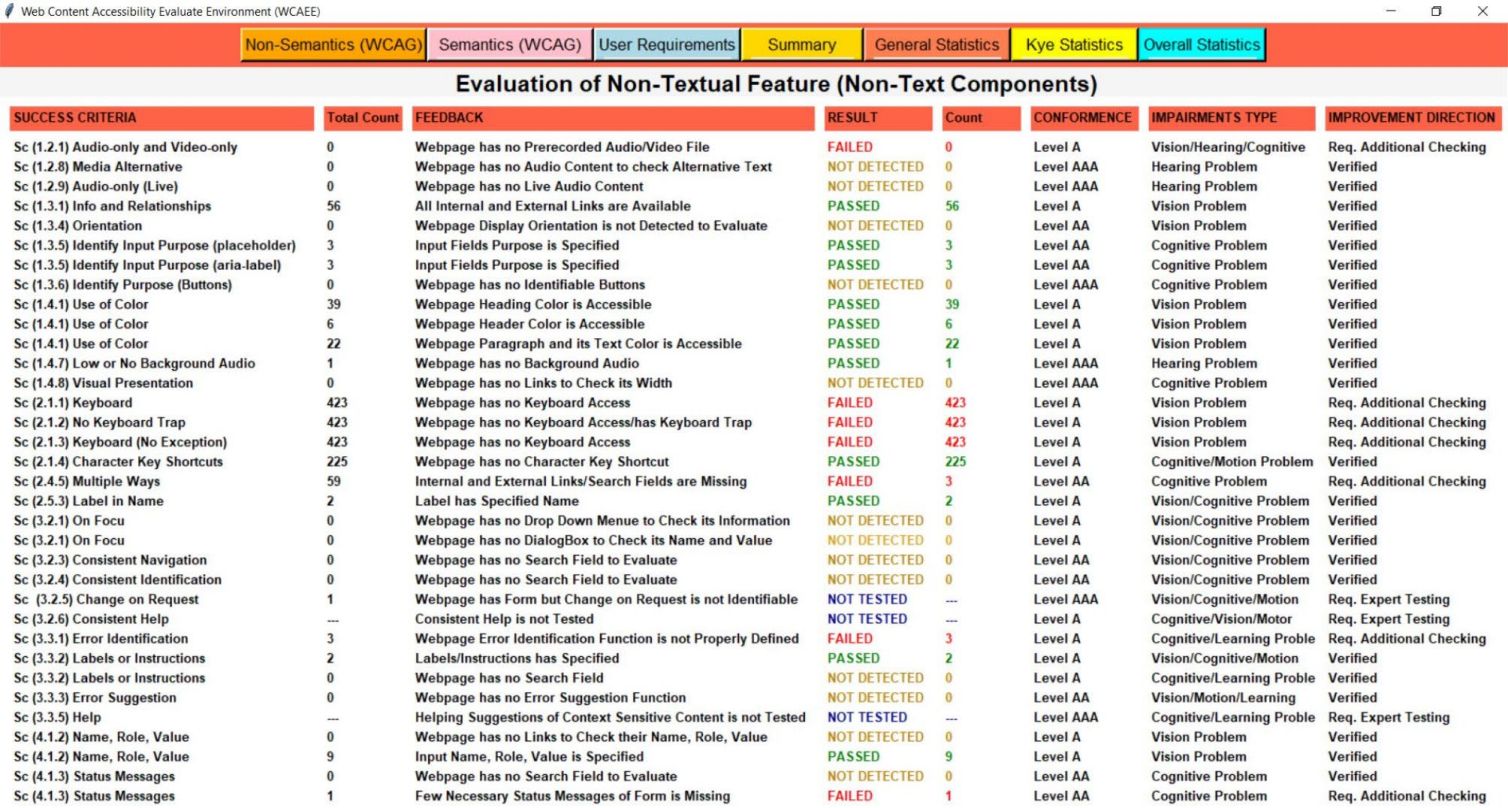
The view of the WCAEE tool for the tested LIVE IT webpage (non-textual elements).

**Fig. 5:**
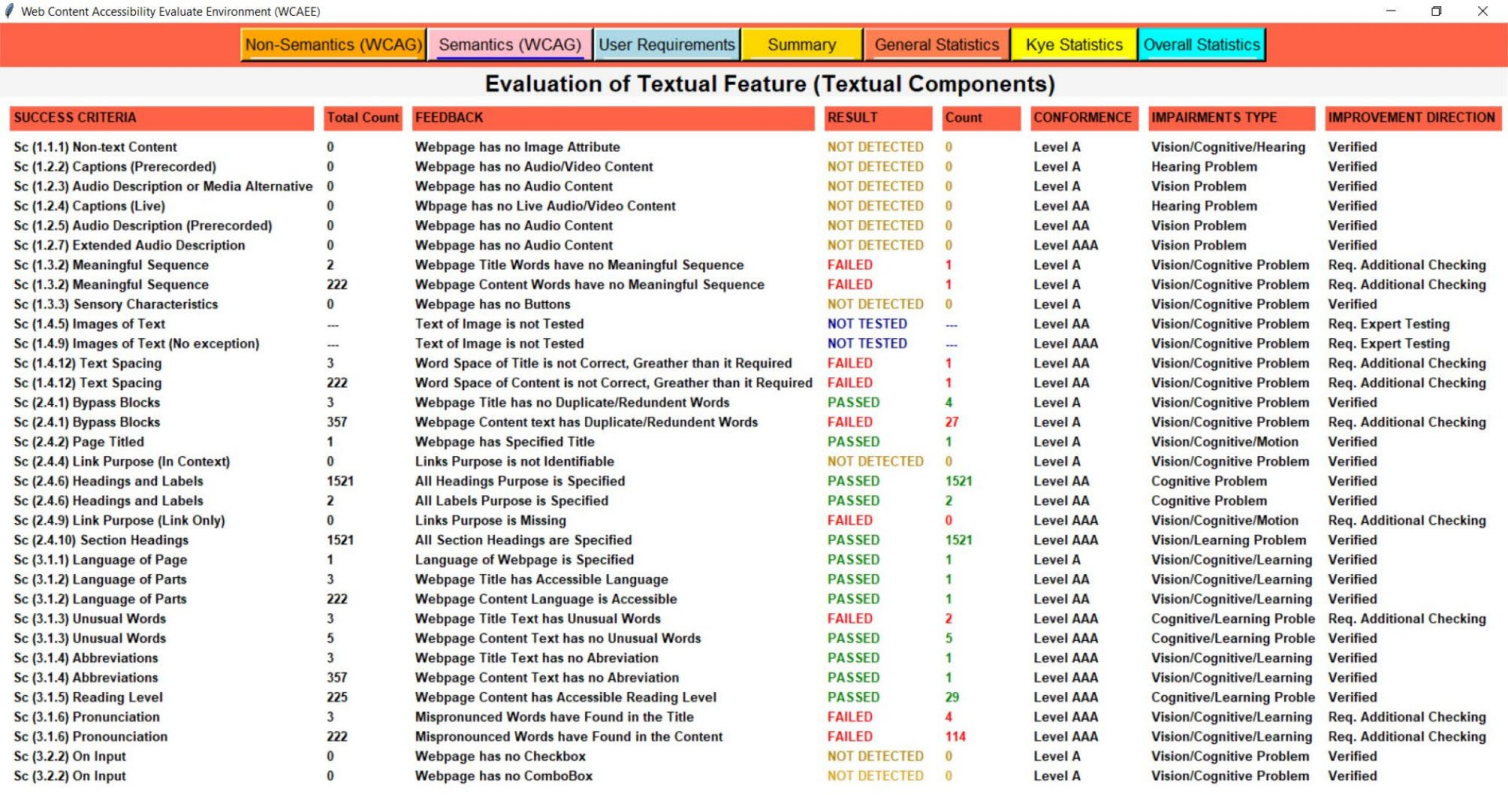
The view of the WCAEE tool for the tested LIVE IT webpage (textual elements).

**Fig. 6:**
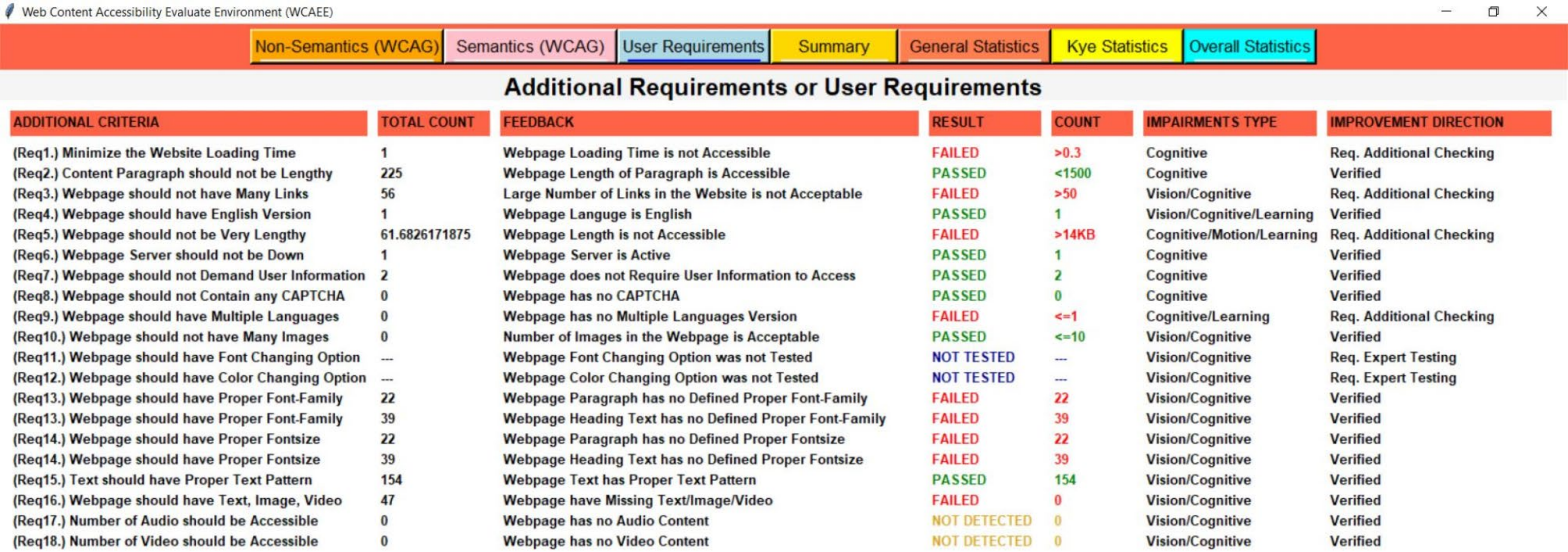
The view of the WCAEE tool for the tested LIVE IT webpage (additional elements).

After applying the three algorithms and performing the complexity analysis, the results of Algo.1 and Algo.2 are organized into eight different aspects through eight columns with titles ‘Success Criteria’, ‘Total Count’, ‘Feedback’, ‘Result’, ‘Count’, ‘Conformance Level’, ‘Impairments Type’ and ‘Improvement Direction’ (as shown in Figs. 4 and 5). Besides, Algo.3 is structured into seven different aspects similar to Algo.1 and Algo.2 except for the Conformance level as shown in Fig. 6. To classify conformance levels based on user requirements is difficult and may be inaccurate because it necessitates the involvement of accessibility professionals and a high degree of accessibility expertise. Figure 1 illustrates how the formulation of the assessment report is carried out via four distinct pages or window views, which are as follows:

**Non-semantic**,** Semantic**,** and Additional Requirement Page**: The output of the Non-Text Complexity Analysis Algorithm, Text Complexity Analysis Algorithm, and Additional Criteria Validation Algorithm for a tested webpage: https://liveit-project.eu/, is displayed on the non-semantic, semantic, and user requirement page, which is represented by Fig. 4, 5, 6. As shown in these figures, to structure and represent the output of each algorithm or evaluation result, the computed complexity result is first presented to the user by structuring with particular WCAG 2.2 success criteria that are relevant to or refer to the particular web objects (first column). In the second, we displayed the total quantity of the particular items that were counted throughout the assessment procedure (second column). After that, we gave feedback regarding the evaluation status (third column). The evaluation result was then given in terms of PASSED, FAILED, NOT DETECTED, and NOT TESTED along with the number of objects that aligned with the result among the total counted objects (fourth and fifth column). To increase the effectiveness of the evaluation report, we have also provided information on the conformance level with reference to the specific success criteria in the sixth column, as well as information on the types of impairments. This information indicates which group of individuals with specific needs these particular success criteria are important to ensure that the web content is accessible to them (seventh column). It helps the user (both designer, developer, and end-user) to understand the importance of each success criterion. In the last, we offered textual improvement directions that show which criteria the tool successfully validated and which criteria need further verification or expert testing. It is recommended to do additional verification for FAILED success criteria and expert testing for NOT TESTED criteria.

**Fig. 7:**
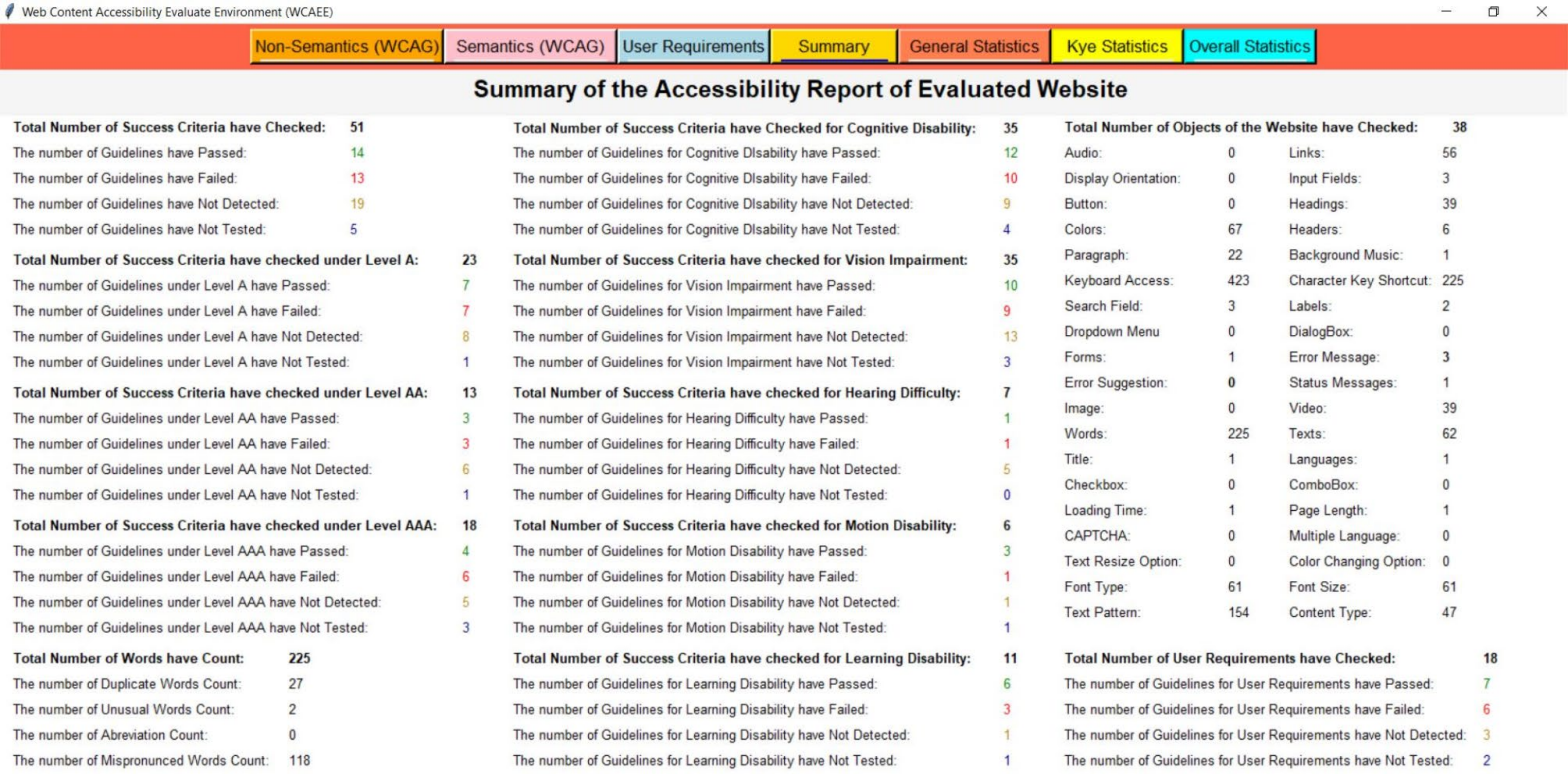
The view of the WCAEE tool for the tested LIVE IT webpage (evaluation summary).

Moreover, Figs. 4, 5, 6 depicts that **Algo.1** (Non-Text Complexity Analysis Algorithm) can evaluate 27 success criteria (1.2.1; 1.2.8; 1.2.9; 1.3.1; 1.3.4; 1.3.5; 1.3.6; 1.4.1; 1.4.7; 1.4.8; 2.1.1; 2.1.2; 2.1.3; 2.1.4; 2.4.5; 2.5.3; 3.2.1; 3.2.3; 3.2.4; 3.2.5; 3.2.6; 3.3.1; 3.3.2; 3.3.3; 3.3.5; 4.1.2; 4.1.3), **Algo.2** (Text Complexity Analysis Algorithm) can evaluate 24 success criteria (1.1.1; 1.2.2; 1.2.3; 1.2.4; 1.2.5; 1.2.7; 1.3.2; 1.3.3; 1.4.5; 1.4.9; 1.4.12; 2.4.1; 2.4.2; 2.4.4; 2.4.6; 2.4.9; 2.4.10; 3.1.1; 3.1.2; 3.1.3; 3.1.4; 3.1.5; 3.1.6; 3.2.2) and **Algo.3** (Additional Criteria Validation Algorithm) can evaluate an additional 18 criteria which by following it is possible to improve the accessibility of the webpage. Besides, after formulating the evaluation result incorporating three different algorithms, we presented the evaluation result in the form of a summary report as shown in Fig. 7.

**Summary Page**: The evaluation report is presented in the summary page or report along with the total number of success criteria that were validated during the evaluation process, the number of success criteria that were checked against three different conformance levels (level A, level AA, and level AAA), and the number of success criteria that validated along with their Passed, Failed, Not Detected, and Not Tested ratios for each type of disability (cognitive, vision, hearing, motion, and learning). The summary report includes a total count of words along with the count of duplicate words, uncommon terms, mispronounced words, and counted abbreviations to provide a brief overview of the evaluated webpage. Additionally, the number of evaluated objects is displayed along with the occurrence number of each object. Besides, the total number of evaluated user requirements have presented with their individual Passed, Failed, Not Detected, and Not Tested ratios. For example, a summary report of a sample of the tested webpage (https://liveit-project.eu/) is shown iFig. . 7. It indicates that the developed tool assessed the tested webpages based on 51 WCAG 2.2 success criteria, of which 14 were successfully Passed, 13 Failed, 19 were found to be Not Detected, and 6 were Not Tested. Furthermore, in terms of conformance level, out of the 51 success criteria that were assessed, 23 were found under level A, 13 under level AA, and 18 under level AAA. Regarding the different types of disabilities, among the success criteria that were tested, 35 related to people with cognitive disabilities and vision impairments, respectively, 7 to people with hearing impairments, 6 to people with motion disabilities, and 11 to people with learning disabilities. Besides, focusing on the tested objects and counted words, 38 objects or features were tested, and 225 words were found on the evaluated webpage. Additionally, 18 criteria were evaluated from the user perspective along with the web content accessibility guideline where for the tested webpage 7 were marked as Passed, 6 as Failed, 3 as Not Detected, and 2 as Not Tested.

##### Evaluation report visualization

**Fig. 8:**
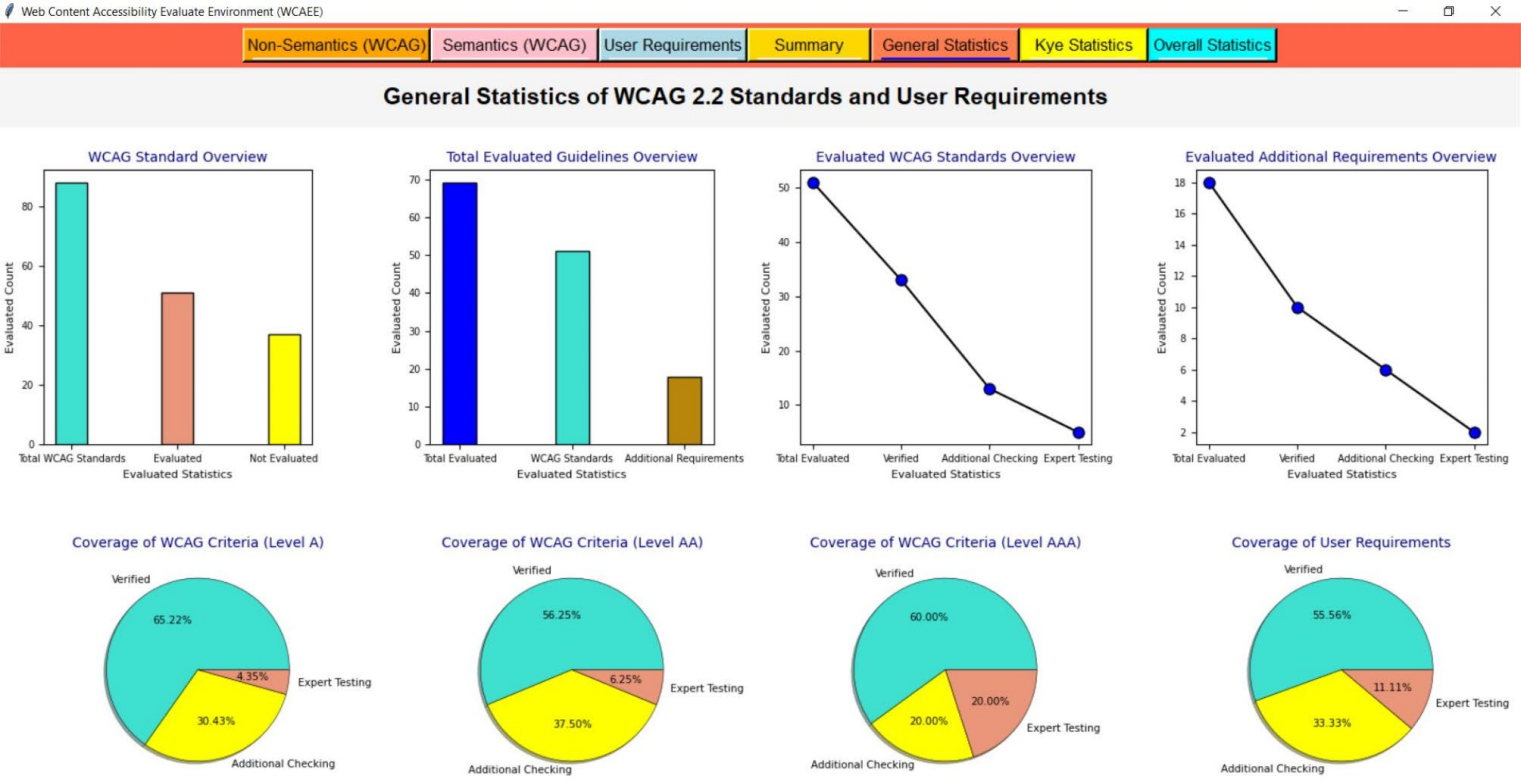
The view of the WCAEE tool for the tested LIVE IT webpage (general statistics).

**Fig. 9:**
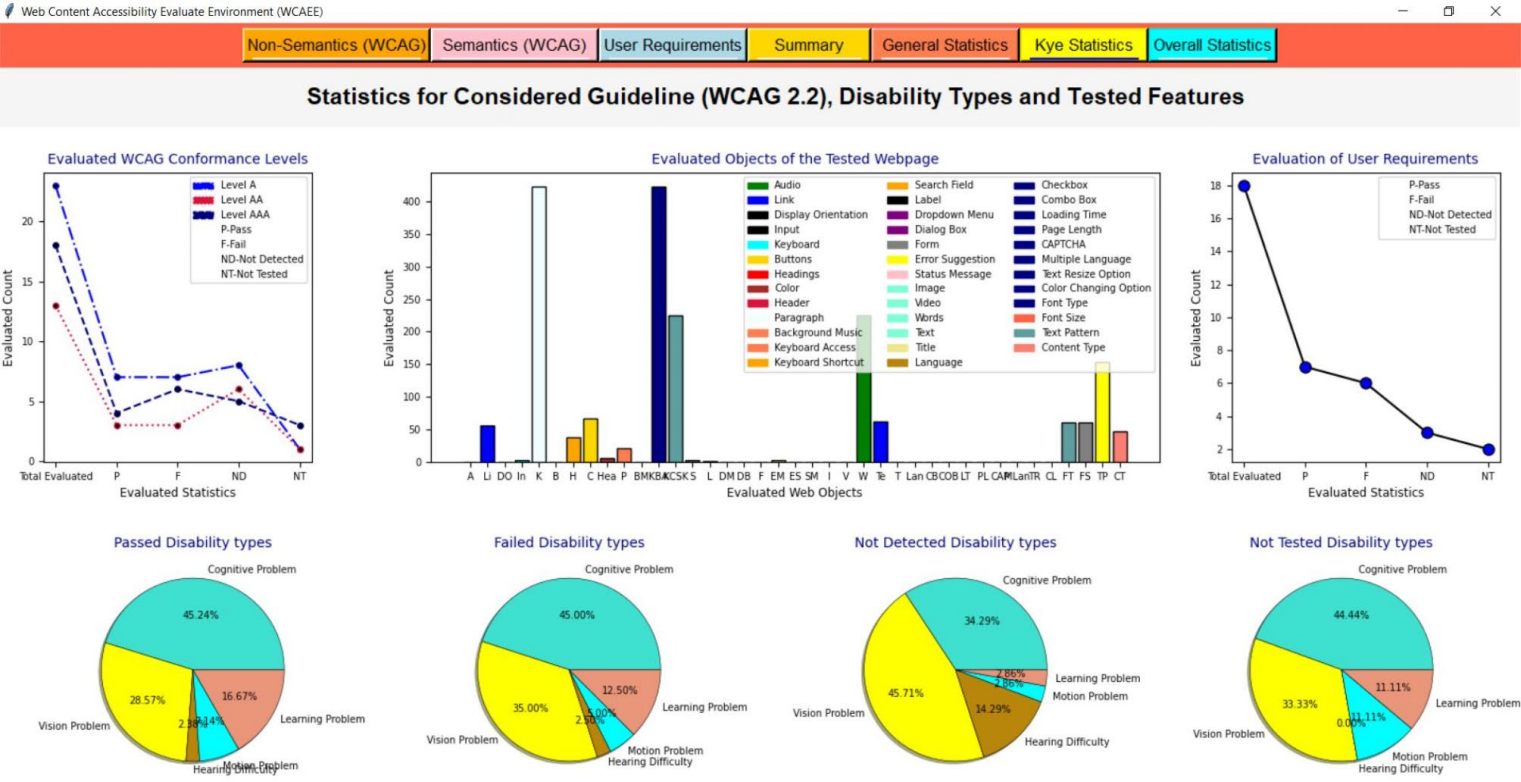
The view of the WCAEE tool for the tested LIVE IT webpage (key statistics).

**Fig. 10:**
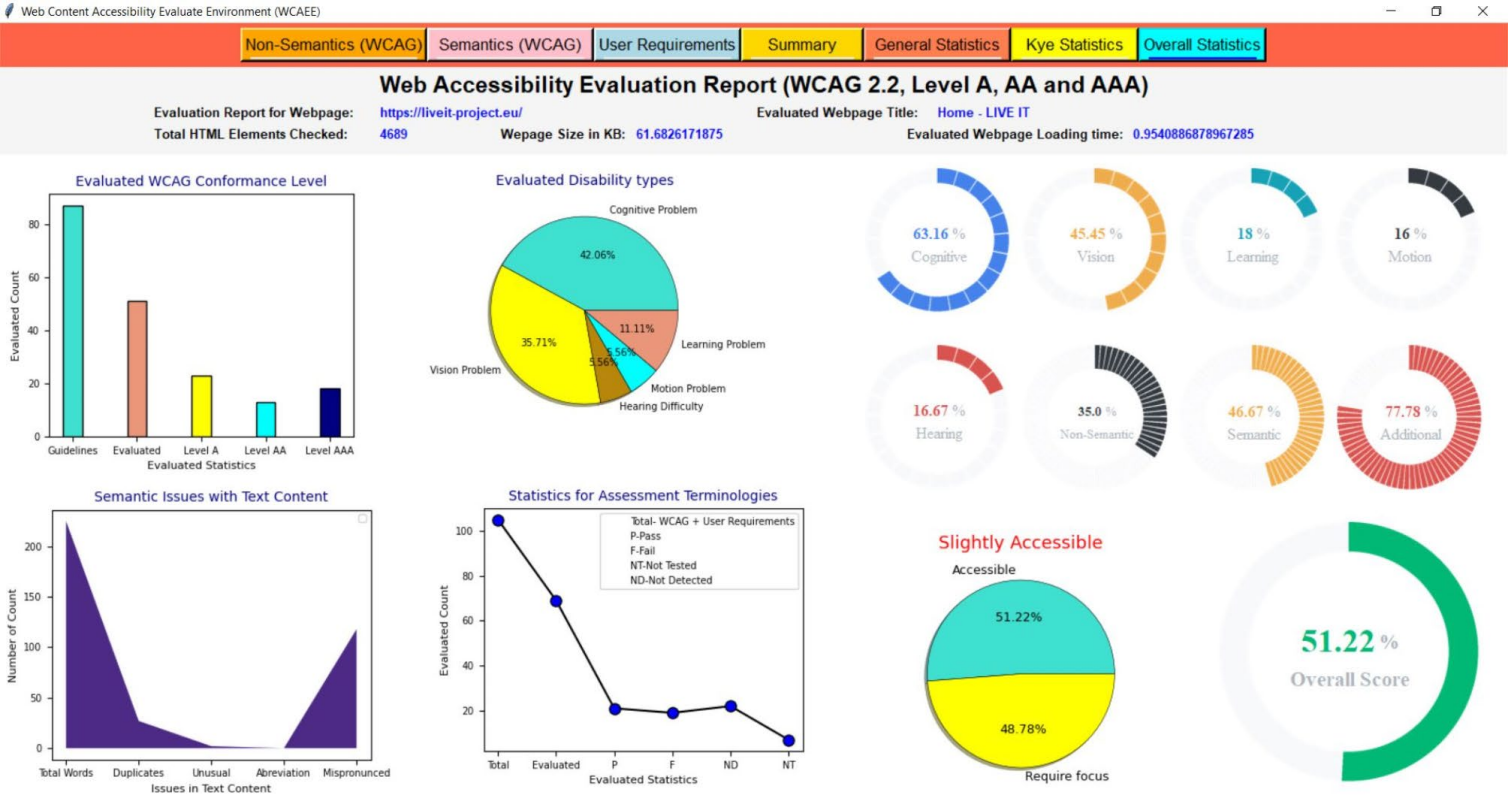
The view of the WCAEE tool for the tested LIVE IT webpage (overall statistics).

After formulating the evaluation report by three different algorithms and providing a summary report, we organized the evaluation result by considering several statistics and displayed the result through multiple graphical representations. For graphical representation, we classified the results into three groups and presented them through three different pages or window views such as ‘General Statistics’, ‘Key Statistics’, and ‘Overall Statistics’ pages which are represented in Figs. 8, 9, 10 and described in the following:

###### General statistic page

In the general statistics page, we represented the evaluation result in terms of evaluated and not evaluated WCAG, the total number of criteria from both WCAG and user requirements that have been considered in the evaluation process, the number of success criteria and user requirements those have evaluated successfully and require additional checking and expert testing in further. Also, in the general statistics report, we provided the coverage ratio of successfully evaluated success criteria along with other success criteria that require additional checking and expert testing from WCAG in terms of three conformance levels. Also, the coverage of additional requirements or user requirements is presented on the general statistics page. For example, Fig. 8 shows the general statistics of the tested webpage formulated by the developed WCAEE tool where all the statistics are represented through several bar charts, line graphs, and pie charts.

In Fig. 8, the bar chart in the upper left corner represents the number of success criteria that have been evaluated and not evaluated through the tool for the tested webpage in accordance with the total number of success criteria in WCAG. This chart shows that out of the 87 WCAG success criteria, 52 can be evaluated automatically by the developed tool, while the remaining criteria need to be observed manually. The second bar graph from the upper left represents the total number of evaluated criteria including WCAG and user requirements. This chart shows that the tool can evaluate 69 criteria total from both WCAG and additional requirements where 51 from WCAG and 18 from user requirements. Similarly, two-line graphs from the upper right corner represented the evaluated criteria (WCAG and additional requirements) in terms of verified, additional checking, and expert testing. It indicates that for both WCAG and user requirements, the highest number of success criteria was able to be verified indicating that the tool can assess these guidelines automatically. However, a few success criteria require further verification and expert testing in addition to the automatic review. Furthermore, the coverage ratio of evaluated criteria (WCAG and additional requirements) in terms of three evaluation statuses (verified, additional checking, and expert testing) is represented by the first three pie charts from the lower left corner in the lower portion of Fig. 8. It shows that, for WCAG, the greatest number of success criteria that are automatically assessed under conformance level A is roughly 65.25%. Also, a significant portion of the success criteria identified as needing further investigation under conformance level AA is around 37.50%. Furthermore, a significant portion of the success criteria that require expert testing under conformance level AAA is roughly 20%. On the other hand, a high percentage of the criteria (about 55.56%) for the user requirements could be automatically assessed and effectively validated by the tool. These statistics help users with a basic understanding of the webpage’s statistics in relation to the targeted success criteria, which aids in the comprehension of their future modification plan to enhance accessibility scenarios.

###### Key statistic page

On the key statistics page, the evaluation result is represented according to the conformance level from WCAG along with user requirements in terms of assessment terminologies (Pass, Fail, Not Detected and Not Tested), statistics of each evaluated web object or features that helps in understanding which types of objects have been considered during the evaluation process, and ratio of each disability type in terms of success criteria that have passed, failed, not tested and not detected. For example, Fig. 9 shows the key statistics of the tested webpage that have been evaluated through the developed WCAEE tool where all the statistics are represented through several line graphs, bar charts, and pie charts.

In Fig. 9, the line graph in the upper left corner represents the evaluated success criteria from WCAG for each conformance level (level A indicated through the blue line; level AA indicated through the red line; level AAA indicated through the navy blue line) in terms of four evaluation/assessment terminologies such as Passed (indicated as P), Fail (indicated as F), Not Detected (indicated as ND) and Not Tested (indicated as NT). It depicts that, for the tested webpage, a significant number of success criteria have passed, failed, and Not Detected for conformance level A while the maximum number of Not Tested success criteria was found for conformance level AAA. Similarly, the line graph in the upper right corner represents the statistics of user requirements in terms of four assessment terminologies. It indicates that for that particular tested webpage, the majority of the requirements have Passed but a significant number of criteria have also failed that direct sufficient improvement is required in the future. The bar graph (in the middle) shows the frequency of each evaluated object on the tested webpage which helps the user in understanding which objects or features have been evaluated during the evaluation by the WCAEE tool. In the lower part of Fig. 9, four pie charts represent the ratio of each type of disability associated with the evaluated guidelines and additional requirements in terms of four assessment terminologies. The first pie chart (from the lower left corner) shows the highest number of criteria passed associated with cognitive problems whereas the second pie chart shows that the failure ratio was also greater for cognitive problems. Besides, the third pie chart shows that the majority of the vision problem-related criteria were marked as not detected which means that these criteria were not implemented in the evaluated webpage or the examined webpage did not use them. Finally, the fourth pie chart represents the not-tested criteria, which was significant in relation to cognitive issues. These four pie charts with the presented ratio will help user comprehend the accessibility scenario of their tested webpage according to each form of impairment.

###### Overall statistic page

In the overall statistics page as represented in Fig. 10, we summarized some arbitrary information that helps to understand some basic information about the tested webpage such as page URLs, page title, total number of checked HTML elements, page size or length, and page loading time. Besides, the major statistics also concluded in the overall statistics page such as evaluated success criteria in terms of each assessment terminologies, overall coverage of each disability type, statistics of several textual types, guidelines overview accordance with conformance level, accessibility score for each disability type, final accessibility status and overall accessibility score. All the statistics are represented through line graphs, pie charts, area charts, bar charts, and progress bars.

The assessment report of the tested webpage and the overall data are depicted in Fig. 10 in terms of evaluated criteria (guidelines and user criteria). The evaluated criterion overview is presented in the upper left corner by the bar graph and pie chart, while the assessment result of the tested webpage is represented by other graphs. The bar graph shows that among the evaluated success criteria from WCAG, most of the WCAG success criteria that were analyzed came from conformance levels A and AAA. Next, the pie chart shows that the majority of the criteria (WCAG success criteria and user criteria) found in the tested webpage are related to issues with cognitive disabilities and vision impairments which are around 42.06% and 35.71% of the total evaluated criteria, respectively. The area chart viewed from the lower left corner reveals that the tested webpage has several mispronounced and duplicated words or terms that seriously impair accessibility for those with cognitive disorders. Additionally, the line graph demonstrates that, although the pass and fail criteria were found to be roughly equal, a significant number of criteria were identified as Not Detected in the tested webpage. It indicates that there are a number of missing or erroneous requirements on the tested webpage that should be taken into account in the future to improve accessibility. After that, eight different progress bar charts showed the accessibility scores according to each type of disability (five types of disabilities), non-semantic (non-textual features), semantic (textual aspects), and additional criteria (user requirements). It shows that the tested webpage has the greatest level of accessibility concern for users with cognitive and vision impairments and the lowest level of concern for users with learning, hearing, and mobility issues. Additionally, the majority of the semantic test criteria have passed, indicating that the tested webpage is more accessible for semantic objects than for non-semantic objects. Besides, the accessibility score (77.78%) was prominent for the criteria related to the user requirements.

Finally, from the left lower part, the progress bar presents the computed overall accessibility score in percentage and the pie chart represents the accessibility status with the accessibility percentage along with the ratio of further improvement. Besides, to identify the accessibility status, we categorized the overall accessibility score according to four ratios: ‘*Accessible’ if (OverallAccessibilityScore* ≥ *90); ‘Comparatively Accessible’ if (OverallAccessibilityScore* ≥ *80); ‘Partially Accessible’ if O(verallAccessibilityScore* < *80 && OverallAccessibilityScore* ≥ *60); and ‘Slightly Accessible’ if (OverallAccessibilityScore* < *60)*. Furthermore, the overall accessibility score is computed through Eq. 1. For the tested webpage, the overall accessibility score was 51.22% which indicates that the tested webpage has significant accessibility issues that require sufficient improvement in the future.

1 $$Overall\,Accessibility\, Score=\frac{Total\, passed}{Total \,failed\,+\,Total\, not \,detected}*100$$

### WCAEE tool evaluation and validation methodology

Some previous works claimed that accessibility issues of webpages depend on a wide array of web objects^36,42^. By considering those web objects in the accessibility evaluation process, it is possible to improve the accessibility status of the current web and provide a comprehensive accessibility evaluation report to the practitioners. Thus, to evaluate and determine the effectiveness of the accessibility evaluation report and computed accessibility score using the developed WCAEE tool, the first objective is to perform an experimental evaluation considering user participation. Following that, to validate the advancement of the developed tool in terms of interactive functionalities, the second objective is to perform a comparative assessment considering functional properties with exiting scientific models and open-source automated tools. The following subsections presented the entire evaluation and validation process in detail.

#### WCAEE tool evaluation

To evaluate the developed WCAEE tool, we conducted a two-phase experimental evaluation. At first, a user-centric evaluation was performed considering user participation where users were asked to assess the sample webpages and rate them based on how well they understood the given criteria. Second, the same sample of webpages is evaluated through the proposed tool to evaluate and compute their accessibility score automatically. Furthermore, based on the user-given ratings, a comparative evaluation has been conducted to evaluate the computed score using the WCAEE tool. Moreover, the prime goal of this experimental evaluation is to evaluate the effectiveness of the computed results generated by the proposed tools in terms of the user perspective.

##### Methods

In the user-centric study, at first, the participants were asked to rate a sample of twenty (20) webpages based on their understanding or preference in terms of accessibility perspective. Second, the same sample of webpages was evaluated through our developed WCAEE tool to formulate their accessibility report. Then, user ratings were further compared with the accessibility scores computed by the WCAEE tool for the same sample of 20 webpages. The study was approved by the local ethics committee of the university of Pannonia.

##### Participants

The user-centric study was conducted by inviting students (bachelor, master, and PhD) from the Department of Electrical Engineering and Information Systems, and the Department of Mathematics, University of Pannonia to test the sample webpages. The experiment was announced and shared in multiple Facebook study groups as well as the university’s official Facebook page to invite students with a Google form registration link where students could write their names, preferred times, and suitable dates that they want to participate in the experiment. The experiment was conducted at the university building in the computer programming lab in the desktop version. Fifty (50) students initially verified their registration, but, seven (7) later withdrew from the test. Therefore, the test was performed by including 43 students which took 28 days long. However, among 43 students, 30 were in the 20–23 age range, 11 were in the 24–27 age range, and 2 were in the 28–year age range. Sixteen (16) of them were foreign students, and the remaining students were Hungarians. Later, three (3) students were removed from the evaluation because they were unable to attend the test due to personal reasons. All the participants reported that they are active on the internet platform, and they use the internet almost every single day for their daily activities. 98% of them mentioned that they use the web for study, 60% for e-commerce portals for online purchasing, and 88% for chatting. Most participants use the internet for more than 30 h a week, and none report using it for fewer than 8 h. From their feedback, it is acceptable that all of the participants are familiar with the internet platform for every kind of browsing which increases the sample’s generalizability. Each participant provided written informed consent attesting to their involvement and granting permission for the data to be used for study and publication purposes.

Furthermore, for evaluating the same sample of selected webpages through the proposed/developed WCAEE automated tool, two PhD students from the Department of Computer Science, University of Pannonia were invited to participate in this experimental evaluation. Both selected students are final-year students and have a high level of understanding about software protocols, and Internet platforms and they have experience with other existing automated web accessibility evaluation tools for their research purposes. As they were already familiar with similar types of evaluation tools, thus it assures their ability to perform the evaluation effectively. However, similar to the user-centric evaluation, we shared all the resources with the two PhD students and invited them to participate in the experiment in the 3D Virtual and Digital Reality Research Laboratory, Department of Electrical Engineering and Information Systems, University of Pannonia. For the declaration, none of the international participants were invited for the experiment due to a limited financial budget.

##### Materials

The most popular top 20 Hungarian university webpages were selected for their accessibility assessment by the user and the developed WCAEE tool. In Table C.1 (Table 2; Appendix C), we listed the selected webpages with URLs that were used in this study. To include in this study, we only considered the English version of the selected webpages as many of our participants were international students (who are staying in Hungary for their studies) and all of the Hungarian students have proficiency in English. Besides, the URLs of the selected page were taken for the homepage only as our developed WCAEE tool supports single-page validation. Thus, our aim is to evaluate and screen the homepage only to balance the result between the user rating and the computed result by the tool.

##### Design and procedure

For the user-centric study, we created comprehensive guidelines on how to perform the evaluation process, what to evaluate, and how the assessment score should be determined based on what criteria govern the user evaluation process. For the instruction guideline, at first, we performed 15-min consultations with the participants before starting the experiment to give them a clear indication of the procedure. To make the experiment effective and less time-consuming, we prepared a list with participants’ names and the associated webpage link that they had to evaluate. In that case, before starting the experiment, we shared the file with the evaluation criteria and scoring instructions prior to initiating the experiment with the user. In this shared file, we divided the forty participants into two groups, with 20 people in each group. To obtain two evaluation scores for a single webpage from distinct participants, we asked each group member to assess two webpages. As a result, 40 participants rated 20 webpages twice, yielding two rating scores. To evaluate, we requested that the user spend a minimum of fifteen minutes on the webpage, during which they were to examine each object and assess its functionality in terms of i) whether or not each web object is executable; ii) how the webpage is represented in terms of color, theme, structuring style, and content (text, image, and video/audio content); iii) whether or not any prototypes have been found that would indicate if the information is missing; and iv) whether or not the information provided is informative. According to the usefulness/importance/availability of the evaluated content, we asked them to submit a score on a 1–5 point scale, where 1 represents the lowest score and 5 represents the best score or highest relevancy with accessibility.

For the WCAEE tool-centric evaluation, before beginning the experiment, we had 20-min discussions with two participants to make sure they understood the experimental process. We gave the user access to the file containing the evaluation guidelines before beginning the experiment. Two PhD student had to perform the experiment for all the selected webpages and report their results. For reporting the results, we ask the participants to report based on three criteria i) whether any webpages are unavailable or not detectable through the WCAEE tool, ii) during the experiment, whether they found any error in the evaluation results and iii) whether all the computed and determine statistics including overall accessibility score is understandable. The entire experiment took an average of 42 min to evaluate and report the results of 20 Hungarian university webpages through the WCAEE tool.

##### User rating validation

As we said in the preceding section, we invited each user from two groups to assess two distinct webpages, thus each page was scored twice by two different participants. As a result, as indicated in Table 4, we receive two ratings for a single page from two distinct participants. For user rating validation, our aim is two-fold, first, (1) evaluate the correlation between two scores (Score A and Score B) for a single webpage to determine how consistent and correlated the user-given score is, and second, (2) carry out a reliability test to determine whether two scores are significantly and positively related to one another. We calculated the average of scores A and B to find the best possible score between the two scores to perform the consistency and reliability test.

**Table 4:** The accessibility scores generated by the participants (Score A, Score B, and Average Score) with classified accessibility status for tested webpages.

Page ID	Score A (group-1)	Score B (group-2)	Avg. score	Accessibility status
1	3.45	3.87	3.66	Partially accessible
2	2.91	3.54	3.225	Partially accessible
3	3.13	3.15	3.14	Partially accessible
4	2.15	2.30	2.225	Slightly accessible
5	3.05	3.15	3.1	Partially accessible
6	1.2	1.50	1.35	Slightly accessible
7	2.78	3.0	2.89	Partially accessible
8	3.65	3.22	3.435	Partially accessible
9	2.97	2.86	2.915	Partially accessible
10	3.82	3.92	3.87	Comparatively accessible
11	2.60	2.64	2.62	Slightly accessible
12	2.59	2.51	2.55	Slightly accessible
13	3.01	2.96	2.985	Partially accessible
14	2.39	2.53	2.46	Slightly accessible
15	1.63	2.55	2.09	Slightly accessible
16	3.0	2.68	2.84	Partially accessible
17	1.59	2.05	1.82	Slightly accessible
18	3.31	3.09	3.2	Partially accessible
19	3.98	3.22	3.6	Partially accessible
20	2.55	2.08	2.315	Slightly accessible

However, the Pearson Correlation Coefficient technique was used to assess the consistency and correlation between Score A and Score B given by the users. The results show that there was a significant positive correlation between the user-rated score A (group 1) and score B (group 2), with Pearson Correlation Coefficients of r (18) = 0.845, and significant at p < 0.001. Besides, the correlation was also evaluated considering the average score with each sample score. It indicates that a significant and positive relationship has been found between Score A, and the average score, with Pearson Correlation Coefficients of *r* (18) = 0.968, and significant at p < 0.001. Also, a significant and positive relationship has been found between Score B, and the average score, with Pearson Correlation Coefficients of *r* (18) = 0.845, significant at p < 0.001. Also, a reliability test was performed through Spearman’s Correlation Coefficients technique. To verify the consistency of the two scores provided by two distinct participants, the correlation between scores A and B was calculated on a single page. With Spearman’s Correlation Coefficients of Rs = 0.8902 and significance at p = 0.001, the bivariate correlation testing shows that there is a substantial correlation between rating score A and rating score B. Besides, we also evaluated the correlation of the average score with each rating score which indicates that a strong and positive correlation was found between Score A and the average score with R_s_ = 0.9534 which is significant at p = 0.001. Also, a strong and positive correlation was found between Score B and the average score with R_s_ = 0.9677 which is significant at p = 0.001. These two statistical analyses depict that the user ratings are normally distributed and significantly correlated with each other which could be included in the final study.

Following a two-step rating correlation analysis of each sample, according to the expert’s opinions (the experts assessed the entire development process, details can be found in Section “Discussion”), we set a threshold score that acts as an indicator for categorizing the accessibility status of the user-provided rating. The threshold value is applicable for the calculated average score as it denotes the optimal score between two ratings or scores. To set the threshold value, we first categorized the accessibility status as Completely Accessible, Comparatively Accessible, Partially Accessible, and Slightly Accessible. We then assigned the threshold value to classify the score where with a score of ≥ 4.5 (90%) denoting Completely accessible, ≥ 3.75 (75%) denoting Comparatively accessible, ≥ 2.75 (55%) denoting Partially accessible, and < 2.75 (< 55%) denoting Slightly accessible. However, we categorized and represented each page’s accessibility status based on the specified threshold value, as indicated in Table 4. It depicts that according to the user opinion; the majority of the pages are partially accessible, and few are slightly accessible. This indicates that the tested webpages have serious accessibility issues that hinder their complete access.

##### WCAEE tool rating validation

To evaluate the developed WCAEE tool rating/score, we assessed the selected webpages (listed in Table C.1 Appendix C) by the proposed or developed tool and represented their computed accessibility score in Table 5. To assess the webpage through the WCAEE tool, we invited two participants to assess every distinct webpage separately, thus each page was experimented with twice by two different participants. As a result, we received two ratings (scores A and score B) returned by the WCAEE tool from two participants for a single page from the two-time experimentation. After that, we calculated the average of scores A and B to find the best possible score between the two scores to categorize their accessibility status. We performed the same experiment at two different times as webpages are continuously updated (either adding or removing the functionalities) which can lead to computing different accessibility results at different times using the same automated testing tool. Thus, one month after the first experiment, the second experiment was conducted to observe whether any differences were found in the evaluation results and whether the proposed tool is able to validate the webpages if there are any changes or modifications in the webpage source code. Table 5 shows that in two different time experiment results, a slight difference has been observed in the few webpages accessibility results that also refers to the ability of the proposed tool to evaluate properly.

**Table 5:** The accessibility scores computed by the WCAEE tool (Score A, Score B, and Average Score) with classified accessibility status for each tested webpage.

Page ID	Score A	Score B	Avg. score	Accessibility status
1	58.97%	58.97%	58.97%	Partially accessible
2	55.0%	52.5%	53.75%	Slightly accessible
3	58.97%	58.80%	58.88%	Partially accessible
4	51.22%	50.09%	50.65%	Slightly accessible
5	51.22%	52.13%	51.67%	Slightly accessible
6	40.91%	42.09%	41.5%	Slightly accessible
7	40.89%	40.88%	40.88%	Slightly accessible
8	58.97%	58.97%	58.97%	Partially accessible
9	55.0%	54.05%	54.52%	Slightly accessible
10	63.16%	63.16%	63.16%	Partially accessible
11	40.91%	40.91%	40.91%	Slightly accessible
12	43.18%	43.18%	43.18%	Slightly accessible
13	67.57%	67.57%	67.57%	Partially accessible
14	43.0%	44.0%	43.5%	Slightly accessible
15	36.96%	35.90%	36.43%	Slightly accessible
16	65.79%	65.79%	65.79%	Partially accessible
17	46.51%	46.51%	46.51%	Slightly accessible
18	63.16%	63.16%	63.16%	Partially accessible
19	44.19%	44.19%	44.19%	Slightly accessible
20	36.96%	36.93%	36.94%	Slightly accessible

To categorize the evaluation results, similar to Table 4, we classified the computed average accessibility scores through sated threshold value into four statuses: Completely Accessible, Comparatively Accessible, Partially Accessible, and Slightly Accessible. Completely Accessible is denoted by a score of greater than or equal to 90% (≥ 90%); Comparatively Accessible is denoted by a score of greater than or equal to 75% (≥ 75%); Partially Accessible is denoted by a score of greater than or equal 55% (≥ 55%); and Slightly Accessible is denoted by a score of less than 55%. These statuses are also represented in Table 5. From this statistic, Table 5 depicts that majority of the tested webpages are categorized as Partially Accessible, and Slightly Accessible. This indicates that none of the webpages are completely accessible which hinders the consistent accessing opportunities for people with disabilities that violates the accessibility criteria.

##### Comparative evaluation (user rating Vs WCAEE rating)

After evaluating the selected webpages from both the user point of view and the developed WCAEE tool, our aim is to perform a comparative evaluation among both tested results to determine their correlation. According to the computed statistic, we evaluated the correlation between the average score obtained from the user study and the computed score by the proposed model. This comparative evaluation helps to determine whether any similarities are observed between two ratings to determine the alignment of user perception with the delivered results computed by the WCAEE tool. Both user’s rating score and score computed by the WCAEE tool with their accessibility status are presented in Table 6. Table 6 shows that most of the tested webpages are categorized as slightly accessible and partially accessible according to both user-provided ratings and scores calculated by the WCAEE tool. Also, for the majority of the tested webpages, the accessibility score generated by the WCAEE tool is significantly related to the participants’ or users’ ratings in terms of their accessibility status. Besides, there was no significant correlation seen between the computed score and user rating for a few webpages (ID-2, ID-5, ID-7, ID-9, ID-10, and ID-19), with respect to their classified accessibility status. It can happen as the developed tool is performing the evaluation through the computer program or written script that acts following advanced engineering techniques. Thus, human perception might be incorrect to observe some critical criteria such as determination of accessible color, complex words, alternative tags and description of image, etc., those actually declared in the webpage source code and not visible to the user directly. Therefore, for some aspects or criteria, it is difficult to evaluate properly for the end users and often it might not be appropriate like a computer program.

**Table 6:** Experimental results with accessibility status from user-given score and computed score by the WCAEE tool.

Page ID	User evaluation		Evaluation by WCAEE	
	Avg. score of user study (between 0–5)	Accessibility status	Avg. score of computed accessibility Score by WCAEE (in percentage)	Accessibility status
1	3.66	Partially accessible	58.97%	Partially accessible
2	3.225	Partially accessible	53.75%	Slightly accessible
3	3.14	Partially accessible	58.88%	Partially accessible
4	2.225	Slightly accessible	50.65%	Slightly accessible
5	3.1	Partially accessible	51.67%	Slightly accessible
6	1.35	Slightly accessible	41.5%	Slightly accessible
7	2.89	Partially accessible	40.88%	Slightly accessible
8	3.435	Partially accessible	58.97%	Partially accessible
9	2.915	Partially accessible	54.52%	Slightly accessible
10	3.87	Comparatively accessible	63.16%	Partially accessible
11	2.62	Slightly accessible	40.91%	Slightly accessible
12	2.55	Slightly accessible	43.18%	Slightly accessible
13	2.985	Partially accessible	67.57%	Partially accessible
14	2.46	Slightly accessible	43.5%	Slightly accessible
15	2.09	Slightly accessible	36.43%	Slightly accessible
16	2.84	Partially accessible	65.79%	Partially accessible
17	1.82	Slightly accessible	46.51%	Slightly accessible
18	3.2	Partially accessible	63.16%	Partially accessible
19	3.6	Partially accessible	44.19%	Slightly accessible
20	2.315	Slightly accessible	36.94%	Slightly accessible

However, according to the presented statistics in Table 6, we can conclude that the WCAEE tool is able to predict the accessibility score that could align with the participants’ perception and have the potential to predict the accessibility of a specific tested webpage because the majority of the webpage’s accessibility status was similar according to the user-given score and the optimally computed score by the tool.

#### WCAEE tool validation

To validate the effectiveness of the developed WCAEE tool, a comparative assessment has been performed considering several similar existing scientific models and existing commercial open-source automated web accessibility testing tools focusing on different functional properties. We conducted this comparative analysis against the seven current scientific models and ten existing automated open-source tools (Accessibility Checker, AccessMonitor, aCe, AChecker, Bulk Accessibility Checker, MAUVE, Rocket Validator, TAW, WAVE, and Nibbler) that have been referenced in the most recent research as listed in Table 7. The aim of this validation is to determine the significant improvements/functionalities that have been addressed in the developed WCAEE tool that distinguish the developed tool from other existing tools.

**Table 7:** Comparative assessment results of the WAEE tool with existing models and automated tools in terms of functional properties.

References	Assessment features							
	Guideline information			Semantic information	Additional evaluation criteria		Accessibility score computation	
	WCAG 2.2	Success criteria feedback	Conformance level feedback	Textual/non-textual evaluation feedback	User and Expert evaluation feedback	User requirements evaluation	Overall accessibility score	Accessibility score for disability type
**Scientific models**								
Jens Pelzetter^43^	✗ (No)	✓(Yes)	✓(Yes)	✗ (No)	✗ (No)	✗ (No)	✓(Yes)	✗ (No)
Michailidou et al.^25^	✗ (No)	✗ (No)	✗ (No)	✗ (No)	✗ (No)	✗ (No)	✓(Yes)	✗ (No)
Shrestha^44^	✗ (No)	✗ (No)	✗ (No)	✗ (No)	✗ (No)	✗ (No)	✗ (No)	✗ (No)
Boyalakuntla et al.^15^	✓(Yes)	✓(Yes)	✓(Yes)	✗ (No)	✗ (No)	✗ (No)	✗ (No)	✗ (No)
Hilera et al.^45^	✗ (No)	✓(Yes)	✓(Yes)	✗ (No)	✗ (No)	✗ (No)	✓(Yes)	✗ (No)
Ingavélez-Guerra et al.^46^	✗ (No)	✓(Yes)	✓(Yes)	✗ (No)	✗ (No)	✗ (No)	✗ (No)	✗ (No)
Robal et al.^29^	✗ (No)	✗ (No)	✗ (No)	✗ (No)	✗ (No)	✗ (No)	✓(Yes)	✗ (No)
**Commercial open-source automated tools**								
Accessibility Checker	✗ (No)	✗ (No)	✗ (No)	✗ (No)	✗ (No)	✗ (No)	✓(Yes)	✗ (No)
AccessMonitor	✗ (No)	✗ (No)	✓(Yes)	✗ (No)	✗ (No)	✗ (No)	✓(Yes)	✗ (No)
aCe	✗ (No)	✗ (No)	✗ (No)	✓(Yes)	✗ (No)	✗ (No)	✗ (No)	✗ (No)
AChecker	✗ (No)	✓(Yes)	✓(Yes)	✗ (No)	✗ (No)	✗ (No)	✗ (No)	✗ (No)
Bulk Accessibility Checker	✗ (No)	✗ (No)	✗ (No)	✗ (No)	✗ (No)	✗ (No)	✗ (No)	✗ (No)
MAUVE	✗ (No)	✗ (No)	✓(Yes)	✗ (No)	✗ (No)	✗ (No)	✓(Yes)	✗ (No)
Rocket Validator	✗ (No)	✗ (No)	✗ (No)	✗ (No)	✗ (No)	✗ (No)	✗ (No)	✗ (No)
TAW	✗ (No)	✓(Yes)	✓(Yes)	✗ (No)	✗ (No)	✗ (No)	✗ (No)	✗ (No)
WAVE	✗ (No)	✗ (No)	✗ (No)	✗ (No)	✗ (No)	✗ (No)	✗ (No)	✗ (No)
Nibbler	✗ (No)	✗ (No)	✗ (No)	✗ (No)	✗ (No)	✗ (No)	✓(Yes)	✗ (No)
**Proposed automated tool**								
WCAEE tool	✓(Yes)	✓(Yes)	✓(Yes)	✓(Yes)	✓(Yes)	✓(Yes)	✓(Yes)	✓(Yes)

To perform the comparative evaluation, in this functional property-centric study, we chose four criteria comprising several functional properties as assessment features. These criteria are linked to the development and visualization of the evaluation results. The first criterion relates to the guideline information that enhances the fairness of the evaluated outcome, such as the applied guideline with its version, success criteria, and conformance level. The second criterion is related to semantic information extraction that helps to evaluate the web content and provide results in terms of semantic and non-semantic aspects and enhances the evaluated result’s semanticity. The third criterion, which relates to additional evaluation criteria, aids in determining whether evaluated criteria still need user or expert evaluation. Also, it allows consideration of additional evaluation criteria such as user requirements along with the implemented standard evaluation guidelines. This criterion helps to improve the effectiveness of the evaluated result. The fourth criterion is related to the computation of the accessibility score, which allows us to determine both the overall accessibility score and the accessibility score for each type of disability. This is a critical component in enhancing the validity and acceptability of the assessed outcome. Based on these four multi-criteria, from the existing literature, seven (7) concrete scientific models and ten automated tools were investigated that not only presented their model or framework but are also practically developed tools that are implemented as real-world applications for accessibility evaluation of webpages. Furthermore, we compared the features of these selected models and tools with our developed WCAEE tool in terms of its functionalities (described earlier). The comparison results are presented in Table 7.

Regarding the selected scientific models, Table 7 shows that, in terms of guideline information, only one model^15^ provides comprehensive feedback about which guideline they have taken into consideration along with success criteria and conformance level information to perform the evaluation; two other models^45,46^ provide success criteria and conformance level information without mentioning the specific guideline; and the remaining models do not address this information in their evaluation feedback or generated report. Regarding the semantic information which refers to whether the model evaluates webpage content considering semantic aspects or not, none of the tools found that consider semantic aspects during their evaluation process. Besides, we think that it is crucial to provide feedback on the evaluation results for any additional evaluation criteria that need user and expert involvement in further evaluation. This is because some evaluation criteria require both automated testing and subsequent user/expert evaluation. Also, it is a wise decision to consider user requirements into account as an additional criterion in addition to the standard guideline as some issues fall under additional evaluation criteria and are not covered by the specific guidelines. From these two aspects, we evaluated the selected models and surprisingly none of the selected models considered these aspects in their evaluation process. Furthermore, we considered two aspects from the accessibility score computation perspective such as overall accessibility score computation as it helps end users to understand the level of accessibility of the tested webpage and provides accessibility score for each type of disability along with the overall score. Focusing on these perspectives, only three models found those compute the overall accessibility score and none of the models provided an accessibility score for each disability type.

Regarding the selected automated tools, Table 7 depicts that none of the selected tools provides evaluation results against WCAG 2.2, only two tools show the result with success criteria, and four tools consider conformance levels to represent the results. Other tools don’t consider these aspects and summarize the result in a tabular format. From the user’s perspective, the possibility of knowing exactly which guideline is implemented would increase the effectiveness of the evaluated results. All the considered tools do not explicitly provide any information about which checkpoints are not able to be implemented automatically and require additional checking such as manual checking. Thus, the users can’t understand which accessibility criteria are not covered by the tool, and which features have to be manually inspected incorporating user and expert to ensure complete accessibility of the evaluated webpages. Besides, WCAG does not include every aspect that might be difficult for people with disabilities. Thus, other aspects that are not considered in WCAG need to be incorporated into the evaluation process along with WCAG. For example, webpage loading time, webpage appropriate length, and the ratio of arbitrary information such as links, images, forms, etc. which could be obtained from user and expert opinion. Surprisingly none of the selected tools consider such aspects in their evaluation process. In terms of metrics that are used for summarising the identified issues and computed results, four tools provide overall accessibility percentage or ratio of accessibility which helps end users understand how accessible the evaluated web page/site is. However, none of the selected tools represents the accessibility score or percentage in terms of disability type. In such a way, it is not clear enough about how much accessible the evaluated webpage is for a particular disability type.

Moreover, this comparison result indicates that none of the selected models/tools fulfilled all the aspects that have been considered to evaluate their functional properties. Besides, the proposed WCAEE tool considers all the aspects that have been selected for the evaluation. From this, it can be concluded that our developed tool has the ability to evaluate webpages considering several crucial aspects to generate the evaluation report that might facilitate the evaluation process and enhance the evaluation report.

## Discussion

Several studies have described webpage accessibility as a situation where it directly impacts the opportunities for webpage access^1^. A few of the studies also mentioned that mostly visually and cognitively impaired users find that the majority of webpages are very complex from their interaction standpoint^47,48^. Indeed, several studies conducted user studies and have shown that if a page is too complex and becomes inaccessible, then most of the visually and cognitively impaired users will not even try or interact with the web content or might avoid browsing the portal a second time. Also, studies have indicated that it would be beneficial for that specific group of people to save time and maintain consistency in their mental health if it were possible to provide a notice outlining the expected difficulties that visually and cognitively impaired users might encounter when navigating, as well as to indicate irrelevant or less important information and the required concern that needs to be focused before starting the navigation process. To address all of these issues, the prime solution would be to provide complete accessibility to the webpages. To ensure complete accessibility, identification of the accessibility issues could be a great solution that could facilitate the accessibility improvement process. An advanced accessibility evaluation tool can make the accessibility improvement process more sophisticated. Therefore, the prime objective of this paper is to explain the end-to-end work of our developed tool for the accessibility evaluation of webpages. In this paper, we presented an implementation of our accessibility evaluation model in a tool, and a confirmatory user study to validate the evaluation score that is automatically generated by our developed tool.

From the very beginning of the design phase through the development phase, expert assessment is carried out to enhance the efficacy of the developed tool and streamline the development process. This enables the experts to monitor the development criteria and offer their feedback. During the design and development phase, two accessibility experts from the University of Pannonia in Hungary who had more than fifteen years of research experience in the fields of accessibility in digital platforms, software development, interface design, human–computer interaction, and color perspective were involved. We preferred to select the expert from the same faculty where the proposed tool has been developed as the expert’s guidance is a continuous process and it requires consistent support, guidance, observation, and suggestions to successfully achieve the goal. Thus, we believe that selecting the expert from the same faculty is a wise decision and we declare that the development process did not bias by any means. In the design phase, we followed the advice of the experts and designed the tool with six distinct window views, classifying the results differently in each view to improve the effectiveness and user comprehension of the evaluated result representation process. This criterion also distinguished the developed tools from other existing tools. Additionally, experts recommended using graphical representation because it could effectively reflect the evaluation outcome. Also, they suggested considering several accessible colors for the visualization of the statistics.

Our method uses the source code of webpages, which is a very valuable, practical, and useful resource to understand the webpage structure from scratch, in contrast to other approaches^4,49^ that use multiple resources, such as webpage screenshots, an evaluation report from other open sources tools, labeled structured web data, etc. However, there are several approaches are also available in the literature^15,25,29,43–46^ that use several tools and techniques to evaluate the source code of webpages to determine their accessibility, but these approaches have excessive computing time, minimal functional and evaluation properties, higher cost and require multiple resources. In that case, our proposed approach particularly the developed tool is relatively simple, and it is faster in evaluation completion and computing accessibility scores by evaluating the source code of a given webpage.

In this paper, we focus on issues or complexity with the accessibility of webpages, as a webpage might be very simple, but it does not guarantee that it will be free from any accessibility issues or that users will find it completely accessible or not experience any difficulty while navigating the page. Predicting webpage accessibility regarding user perception considering an algorithmic evaluation process is a challenging task as the webpage is a combination of several elements that need into consideration from both visual aspects and structural and design aspects. For example, page type, font type, and sizes, color combination, navigation elements, objects, dynamic content, textual content, visual content, etc. Thus, to develop an updated and improved algorithmic evaluation process, advanced technologies could play a great role. Therefore, we hypothesized that by implementing advanced technologies, we could develop an effective solution to evaluate webpage accessibility. However, some studies conducted to investigate the most important features of the web that act as a prime factor in reduced accessibility. Unfortunately, they came up with a solution agreeing on a non-binary solution which means they didn’t find any optimal answer for the particular research. Therefore, researchers suggested that the more features that could be investigated, the more effective results will be. Therefore, following our hypothesis, in our algorithm, we considered all elements or web objects related to the Web Content Accessibility Guidelines and additional objects from user criteria to evaluate the webpage regarding their impact on the content. However, all the elements are also separated from the perspective of textual/non-textual features. To evaluate both textual and non-textual features, we applied separate algorithms and different techniques. For example, for textual features evaluation, we considered different NLP techniques, and for non-textual features, we considered several auxiliary techniques.

We conducted a confirmatory user evaluation as well as a functional property-based evaluation to verify the evaluation outcome of the proposed tool. In the user evaluation, each user rated each page twice and their ratings were validated in terms of consistency and reliability by observing the correlation coefficient. The correlation coefficient indicates that participants’ evaluations were highly correlated and consistent with their responses. Furthermore, a qualitative assessment has been carried out for every selected page, considering user ratings in conjunction with the accessibility score calculated by the proposed tool. The accessibility score generated by the developed model is qualitatively analyzed, and the results show that, for the vast majority of the tested webpages, the computed score substantially matches user evaluations. Our additional results also showed that few webpages’ user ratings and computed scores by the proposed tool have a lack of similarity. For example, University of Szeged, Széchenyi István University, Semmelweis University, University of Pannonia, and the University of Kaposvár webpage were rated as partially accessible by the participants that show users were quite satisfied with their navigation facilities but according to the computed score by the proposed tool, it rated slightly accessible that means the webpage has serious accessibility issues with its majority of the investigated features. Similarly, Óbuda University was rated as comparatively accessible by users, meaning that its webpage includes highly accessible features. However, the score calculated by the proposed tool classified it as partially accessible, which is inconsistent with the user’s viewpoint. This may occur because the developed tool uses an algorithmic evaluation process to compute the score, taking into account a number of a webpage’s structural elements. Additionally, it identifies certain elements that are typically assigned or hidden in the underlying source code, making them difficult for average users to understand. Besides, the functional property-based evaluation is performed by comparing the proposed tool with the existing scientific models and open-source automated tools considering several properties that are directly related to the improvement of the tool’s effectiveness. This comparative result shows that the proposed tool has high consideration about the evaluation criteria and representation strategies, which helps to understand the accessibility status and features of the tested webpage better than other tools which also directly influences the improvement of the tools’ effectiveness.

From the two-phase assessment, our hypothesis stated that the evaluation result of the proposed tool has a great effect on the improvement of the tool’s effectiveness. Also, the tool’s automatically generated accessibility scores are substantial and strongly correlated with users’ evaluation. It suggested that the tool has the potential for the assessment and computation of webpage accessibility scores. Therefore, according to the two-phase evaluation, it could be concluded that it is feasible to increase the effectiveness of the accessibility evaluation result by considering advanced techniques and assessing both arbitrary and non-arbitrary web features.

Although the proposed tool has the potential to evaluate webpage accessibility, it is not free from limitations. First of all, in our work, we do not consider any dynamic elements and their effect on our evaluation process to compute accessibility scores. Further studies are suggested for a better understanding of the impact of dynamic content on accessibility. Secondly, even though we have quite a large number of ratings around 80 ratings of 40 users on 20 pages, further studies also suggested incorporating more users and more webpages. Besides, due to the financial budget and limited time, at this stage, we validated the proposed tool considering students’ evaluation results, although evaluating incorporating specialists would provide more suitable evaluation results. Thus, this is one of the major limitations of this study that will be considered in our future work. In our studies, we selected Hungarian university webpages, further studies might be conducted with other domain webpages from other countries or the same country. Also, the proposed tool doesn’t offer the downloading option of the evaluation report. Therefore, in our future work, we intend to extend the tool’s functionality by adding the ability to download the evaluation reports. In this scenario, the user can send a request to the server and the server can respond to the request by sending an accessibility evaluation report of the tested page in a variety of file formats that the end users can easily access, such as JSON, Excel, or PDF. Also, in future work, we aim to test the tool by inviting more experts and compare our proposed tool evaluation result with existing open-source tools results considering the same set of data and parameters.

## Conclusion

Generally, issues or problems with accessibility can be defined as “the degree of difficulty in navigating resources of the web platform”, although it is hard to characterize and systematically assess. In this paper, we presented our end-to-end work to provide a tool that can evaluate webpage objects and determine their level of accessibility. Predicting accessibility issues of a webpage might have many applications ranging from supporting accessibility criteria to providing better accessibility evaluation techniques. However, the work presented in this paper is a development and experimental work where we developed and implemented our tool to evaluate accessibility issues and generate accessibility scores for webpages based on common aspects of their HTML Document Object Model (DOM) considering structural and visual elements. In the evaluation report, for each webpage, an accessibility score determines the level of freely accessing opportunities of the particular webpage. To evaluate and validate the generated accessibility score by the tool through our proposed algorithms, a two-fold assessment was conducted. This two-fold assessment supported that the proposed model is functionally enriched with different properties that were found absent in other similar approaches, also the generated accessibility scores that are automatically generated by our tool are strongly correlated with the user’s ratings. Therefore, users can have an initial perception of the accessibility of the tested webpage, and web designers and developers can use this framework to take a complete direction to design and develop an accessible webpage maintaining usability and accessibility criteria. We believe that this work is an important contribution to the area of web accessibility domain because by using this tool, it is possible to represent the evaluation results in an implicit way focusing on every group of users including special needs.

## Electronic supplementary material

Below is the link to the electronic supplementary material.

Supplementary Material 1

## Data Availability

No datasets were generated or analysed during the current study.

## References

[1] Ara, J. & Sik-Lanyi, C. Investigation of Covid-19 vaccine information websites across Europe and Asia using automated accessibility protocols. *Int. J. Environ. Res. Public Health* **19**(5), 2867 (2022). 35270555 10.3390/ijerph19052867 PMC8910771

[2] Laamanen, M., Ladonlahti, T., Puupponen, H. & Kärkkäinen, T. Does the law matter? An empirical study on the accessibility of Finnish higher education institutions’ web pages. *Univ. Access Inf. Soc.* **23**(1), 475–491 (2024). 10.1007/s10209-022-00931-6 PMC9662130 36407565

[3] Shah, H. Enhancing web accessibility-navigating the upgrade of design systems from WCAG 2.0 to WCAG 2.1.

[4] Reinecke, K., Yeh, T., Miratrix, L., Mardiko, R., Zhao, Y., Liu, J., & Gajos, K. Z. Predicting users’ first impressions of website aesthetics with a quantification of perceived visual complexity and colorfulness. In *Proceedings of the SIGCHI conference on human factors in computing systems* (pp. 2049–2058) (2013).

[5] Maphalala, M. C. & Adigun, O. T. Academics’ experience of implementing E-learning in a South African higher education institution. *Int. J. High. Educ.* **10**(1), 1–13 (2021).

[6] Schiavone, A. G. & Paternò, F. An extensible environment for guideline-based accessibility evaluation of dynamic Web applications. *Univ. Access Inf. Soc.* **14**(1), 111–132 (2015).

[7] Wu, O., Hu, W. & Shi, L. Measuring the visual complexities of web pages. *ACM Trans. Web (TWEB)* **7**(1), 1–34 (2013).

[8] López-Gil, J. M. & Pereira, J. Turning manual web accessibility success criteria into automatic: an LLM-based approach. *Universal Access in the Information Society*, 1–16 (2024).

[9] Ara, J., Sik-Lanyi, C., Kelemen, A. & Guzsvinecz, T. An inclusive framework for automated web content accessibility evaluation. *Universal Access in the Information Society*, 1–27 (2024).

[10] Bajammal, M. & Mesbah, A. Semantic web accessibility testing via hierarchical visual analysis. In *2021 IEEE/ACM 43rd International Conference on Software Engineering (ICSE)* (pp. 1610–1621). IEEE (2021).

[11] Miranda, D., Araújo, J. & Liebel, G. A conceptual model for web accessibility requirements in agile development (2024).

[12] Chadli, F. E., Gretete, D. & Moumen, A. Digital accessibility: A systematic Literature Review. In *SHS Web of Conferences* (Vol. 119). EDP Sciences (2021).

[13] Bai, A., Fuglerud, K. S., Skjerve, R. A. & Halbach, T. Categorization and comparison of accessibility testing methods for software development (2018). 30371447

[14] Chiang, M. F., Cole, R. G., Gupta, S., Kaiser, G. E. & Starren, J. B. Computer and world wide web accessibility by visually disabled patients: Problems and solutions. *Surv. Ophthalmol.* **50**(4), 394–405 (2005). 15967193 10.1016/j.survophthal.2005.04.004

[15] Boyalakuntla, K., Venigalla, A. S. M. & Chimalakonda, S.. WAccess–A Web Accessibility Tool based on the latest WCAG 2.2 guidelines (2021). arXiv preprint arXiv:2107.06799.

[16] Pelzetter, J. A declarative model for accessibility requirements. *In Proceedings of the 17th International Web for All Conference (pp. 1–10)* (2020)*.*

[17] Wijaya, I. G. N. S., Triandini, E., Kabnani, E. T. G. & Arifin, S. (2021). E-commerce website service quality and customer loyalty using WebQual 4.0 with importance performances analysis, and structural equation model: An empirical study in shopee. *Register* **7**(2), 107–124.

[18] Okechukwu, N. N. & Anunobi, C. V. Availability and usability of academic library websites by undergraduates in federal universities in South East Nigeria. *UNIZIK J. Res. Lib. Inf. Sci.* **5**(1), 87–100 (2020).

[19] Carvalho, L. P. the epidemiology of web accessibility: Improving internet health by identifying and tackling the root cause of web accessibility issues. *ACM SIGACCESS Access. Comput.* **137**, 1–1 (2024).

[20] ARAa, J. & Cecilia, S. L. Artificial intelligence in web accessibility: potentials and possible challenges. *Proceedings of IAC*, 173 (2022).

[21] Fernandes, N., Costa, D., Duarte, C. & Carriço, L. Evaluating the accessibility of web applications. *Procedia Comput. Sci.* **14**, 28–35 (2012).

[22] Freire, A. P., & de Mattos Fortes, R. P. Automatic accessibility evaluation of dynamic web pages generated through XSLT. In *Proceedings of the 2005 International Cross-Disciplinary Workshop on Web Accessibility (W4A)* (pp. 81–84) (2005).

[23] Ivory, M. Y. & Megraw, R. Evolution of web site design patterns. *ACM Trans. Inf. Syst. (TOIS)* **23**(4), 463–497 (2005).

[24] Rojbi, S., Rojbi, A. & Gouider, M. S. Enhancing the accessibility of images on the web: A holistic view and new perspectives.

[25] Michailidou, E., Eraslan, S., Yesilada, Y. & Harper, S. Automated prediction of visual complexity of web pages: Tools and evaluations. *Int. J. Hum Comput Stud.* **145**, 102523 (2021).

[26] Duarte, C., Matos, I., & Carriço, L. Semantic content analysis supporting web accessibility evaluation. In *Proceedings of the 15th International Web for All Conference* (pp. 1–4) (2018).

[27] Doush, I. A., Alkhateeb, F., Al Maghayreh, E. & Al-Betar, M. A. The design of RIA accessibility evaluation tool. *Adv. Eng. Softw.* **57**, 1–7 (2013).

[28] Bonacin, R., Reis, J. C. D. & de Araujo, R. J. An ontology-based framework for improving color vision deficiency accessibility. *Universal Access in the Information Society*, *1–26* (2021)*.*

[29] Robal, T., Marenkov, J., & Kalja, A. Ontology design for automatic evaluation of web user interface usability. *In 2017 Portland International Conference on Management of Engineering and Technology (PICMET) (pp. 1–8). IEEE* (2017)*.*

[30] Rashida, M. et al. Towards developing a framework to analyze the qualities of the university websites. *Computers* **10**(5), 57 (2021).

[31] Alsaeedi, A. Comparing web accessibility evaluation tools and evaluating the accessibility of webpages: proposed frameworks. *Information* **11**(1), 40 (2020).

[32] Duarte, C., Salvado, A., Akpinar, M. E., Yeşilada, Y. & Carriço, L. Automatic role detection of visual elements of web pages for automatic accessibility evaluation. In *Proceedings of the 15th International Web for All Conference* (pp. 1–4) (2018).

[33] Hall, R. H. & Hanna, P. The impact of web page text-background colour combinations on readability, retention, aesthetics and behavioural intention. *Behav. Inf. Technol.* **23**(3), 183–195 (2004).

[34] Ikhsan, I. N. & Candra, M. Z. C. Automatically: An automated refactoring method and tool for improving web accessibility. In *2018 5th International Conference on Data and Software Engineering (ICoDSE)* (pp. 1–6). IEEE (2018).

[35] Abu Doush, I. et al. Web accessibility automatic evaluation tools: To what extent can they be automated?. *CCF Trans. Pervasive Comput. Interact.* **5**(3), 288–320 (2023).

[36] Mazalu, R. & Cechich, A. Web accessibility assessment through multi-agent support for visually impaired users. *Int. J. Coop. Inf. Syst.* **29**(03), 2050005 (2020).

[37] Campoverde-Molina, M., Luján-Mora, S. & Valverde, L. Process model for continuous testing of web accessibility. *IEEE Access* **9**, 139576–139593 (2021).

[38] Iannuzzi, N., Manca, M., Paternò, F. & Santoro, C. Usability and transparency in the design of a tool for automatic support for web accessibility validation. *Univers. Access Inf. Soc.* **23**(1), 435–454 (2024).

[39] Ara, J., & Sik-Lanyi, C. AccGuideLiner: Towards a modelling approach of web accessibility requirements following WCAG 2.2. In *2023 IEEE International Conference on Smart Information Systems and Technologies (SIST)* (pp. 10–15). IEEE (2023).

[40] Ara, J., Sik-Lanyi, C. & Kelemen, A. Accessibility engineering in web evaluation process: a systematic literature review. *Universal Access in the Information Society*, 1–34 (2023). 10.1007/s10209-023-00967-2 PMC9881526 36721782

[41] Rubano, V. & Vitali, F. Experiences from declarative markup to improve the accessibility of HTML. In *Balisage: The Markup Conference* (pp. 1–22) (2020).

[42] Giraud, S., Thérouanne, P. & Steiner, D. D. Web accessibility: Filtering redundant and irrelevant information improves website usability for blind users. *Int. J. Hum. Comput. Stud.* **111**, 23–35 (2018).

[43] Pelzetter, J. Using ontologies as a foundation for web accessibility tools. In *Proceedings of the 15th International Web for All Conference* (pp. 1–2) (2018).

[44] Shrestha, R. A neural network model and framework for an automatic evaluation of image descriptions based on NCAM image accessibility guidelines. In 2021 *4th Artificial Intelligence and Cloud Computing Conference* (pp. 68–73) (2021).

[45] Hilera, J. R., Otón, S., Martin-Amor, C. & Timbi-Sisalima, C. Towards a service-based architecture for web accessibility federated evaluation. In *9th International Conference on Advances in Computer-Human Interactions (ACHI’16)* (pp. 6–10) (2016).

[46] Ingavelez-Guerra, P., Robles-Bykbaev, V., Oton, S., Vera-Rea, P., Galan-Men, J., Ulloa-Amaya, M. & Hilera, J. R. A proposal based on knowledge modeling and ontologies to support the accessibility evaluation process of learning objects. In 2018 Congreso Argentino de Ciencias de la Informática y Desarrollos de Investigación (CACIDI) (pp. 1–5). IEEE (2018).

[47] Zeboudj, M., & Belkadi, K. Designing a Web accessibility environment for the visually impaired. In *2022 3rd International Conference on Embedded & Distributed Systems (EDiS)* (pp. 154–157). IEEE (2022).

[48] Enco-Jáuregui, L., Meneses-Claudio, B. & Auccacusi-Kañahuire, M. Web accessibility for people with dyslexia: A systematic literature review. *EAI Endorsed Trans. Pervasive Health Technol.* **9** (2023).

[49] Riegler, A. & Holzmann, C. Measuring visual user interface complexity of mobile applications with metrics. *Interact. Comput.* **30**(3), 207–223 (2018).

