# Supplementary material for: Automated evaluation of accessibility issues of webpage content: tool and evaluation
Source: Sci Rep. 2025 Mar 19;15:9516. doi: 10.1038/s41598-025-92192-5 (PMC11923163; doi:10.1038/s41598-025-92192-5)
Supplement: Supplementary file 1 — Supplementary Material 1 [file 41598_2025_92192_MOESM1_ESM.docx]

**Appendix A. Important libraries**

from tkinter import*

from tkinter import messagebox

import os

import tkinter as tk

import tkinter as tkinter

from tkinter import ttk

import urllib.request

from bs4 import BeautifulSoup

import validators

import re

import nltk

nltk.download('punkt')

nltk.download('stopwords')

nltk.download()

from nltk.corpus import words

from nltk.corpus import wordnet

from nltk.corpus import wordnet as wn

from nltk.corpus import stopwords

stopwords.words('english')

stop_words = set(stopwords.words("english"))

from nltk.tokenize import word_tokenize, sent_tokenize

import language_tool_python

import itertools

from readability import Readability

import requests as re

from matplotlib.backends.backend_tkagg import FigureCanvasTkAgg

import matplotlib.pyplot as plt

import matplotlib.patches as mpatches

import requests

import time

from langdetect import detect, detect_langs

from langdetect import DetectorFactory

import language_tool_python

**Appendix B. Guideline implementation direction and complexity analysis algorithms**

Table B.1 Webpages components validation guidelines/directions used to develop the proposed WCAEE tool.

**Table 1:** Implementation directions of each addressed element in the proposed WCAEE tool

| **Elements/Objects** | **Non-Textual Elements** | **Textual Elements** | **Additional Elements** | **Related guidelines/Improvement directions** |
| --- | --- | --- | --- | --- |
| Audio/Video | ✓ |  |  | (1.2.1) Audio-only and Video-only; (1.2.2) Captions (Prerecorded); (1.2.3) Audio Description or Media Alternative (Prerecorded); [WCAG 2.2] |
| Links | ✓ | ✓ |  | (1.3.6) Identify Purpose; (1.4.1) Use of Color; (1.4.8) Visual Presentation; (2.4.4) Link Purpose (In Context); (2.4.9) Link Purpose (Link Only), (4.1.2) Name, Role [WCAG 2.2] |
| Display Orientation | ✓ |  |  | (1.3.4) Orientation [WCAG 2.2] |
| Input field | ✓ |  |  | (1.3.3) Sensory Characteristics; (1.3.5) Identify Input Purpose (placeholder); (2.1.1) Keyboard; (2.1.4) Character Key Shortcuts; (3.3.2) Labels or Instructions [WCAG 2.2] |
| Buttons | ✓ | ✓ |  | (1.3.3) Sensory Characteristics; (1.3.5) Identify Input Purpose (placeholder); (1.3.6) Identify Purpose (Buttons); (2.1.1) Keyboard; (3.3.2) Labels or Instructions [WCAG 2.2] |
| Headings | ✓ | ✓ |  | (1.4.1) Use of Color; (2.4.6) Headings and Labels; (2.4.10) Section Headings [WCAG 2.2] |
| Header | ✓ |  |  | (1.4.1) Use of Color; (2.4.10) Section Headings [WCAG 2.2] |
| Paragraph | ✓ | ✓ |  | (1.3.2) Meaningful Sequence; (1.4.1) Use of Color; (2.4.1) Bypass Blocks; (3.1.3) Unusual Words [WCAG 2.2] |
| Background Music | ✓ |  |  | (1.4.7) Low or No Background Audio [WCAG 2.2] |
| Keyboard access | ✓ |  |  | (2.1.1) Keyboard; (2.1.3) Keyboard (No Exception) [WCAG 2.2] |
| Keyboard Character Key Shortcut | ✓ |  |  | (2.1.4) Character Key Shortcuts [WCAG 2.2] |
| Search Field | ✓ |  |  | (2.4.5) Multiple Ways; (3.2.3) Consistent Navigation; (3.2.4) Consistent Identification; (3.3.2) Labels or Instructions; (4.1.3) Status Messages [WCAG 2.2] |
| Label | ✓ | ✓ |  | (1.3.3) Sensory Characteristics; (1.3.5) Identify Input Purpose (placeholder); (1.4.1) Use of Color; (2.1.1) Keyboard; (2.4.10) Section Headings; (2.5.3) Label in Name; (3.3.2) Labels or Instructions [WCAG 2.2] |
| Dropdown Menu | ✓ |  |  | (3.2.1) On Focus; (3.2.2) On Input [WCAG 2.2] |
| Dialog box | ✓ |  |  | (3.2.1) On Focus; (3.2.2) On Input [WCAG 2.2] |
| Form | ✓ |  |  | (3.2.5) Change on Request; (4.1.3) Status Messages [WCAG 2.2] |
| Status Message | ✓ |  |  | (4.1.3) Status Messages [WCAG 2.2] |
| Error Message | ✓ |  |  | (3.3.1) Error Identification; (3.3.3) Error Suggestion [WCAG 2.2] |
| Error Suggestion | ✓ |  |  | (3.3.3) Error Suggestion [WCAG 2.2] |
| Image |  | ✓ |  | (1.1.1) Non-text Content; (1.4.5) Images of Text [WCAG 2.2] |
| Pre-recorded/Live Audio and Video |  | ✓ |  | (1.2.1) Audio-only and Video-only; (1.2.2) Captions (Prerecorded); (1.2.3) Audio Description or Media Alternative (Prerecorded); (1.2.4) Captions (Live) [WCAG 2.2] |
| Title |  | ✓ |  | (1.3.2) Meaningful Sequence; (1.4.12) Text Spacing; (2.4.1) Bypass Blocks; (2.4.2) Page Titled [WCAG 2.2] |
| Words |  | ✓ |  | (1.3.2) Meaningful Sequence; (2.4.1) Bypass Blocks; (3.1.3) Unusual Words [WCAG 2.2] |
| Webpage Text |  | ✓ |  | (1.3.2) Meaningful Sequence; (2.4.1) Bypass Blocks; (3.1.3) Unusual Words; (3.1.4) Abbreviations; (3.1.5) Reading Level; (3.1.6) Pronunciation [WCAG 2.2] |
| Language |  | ✓ |  | (3.1.1) Language of Page [WCAG 2.2] |
| Checkbox |  | ✓ |  | (3.2.1) On Focus; (3.2.2) On Input [WCAG 2.2] |
| Combo boxes |  | ✓ |  | (3.2.1) On Focus; (3.2.2) On Input [WCAG 2.2] |
| Loading time |  |  | ✓ | Webpage loading time should be <=0.3 seconds |
| Paragraph length |  |  | ✓ | (1.3.3) Sensory Characteristics; (1.3.4) Orientation [WCAG 2.2] |
| Hyperlink ratio |  |  | ✓ | Webpage number of internal and external or hyperlinks must be <=50 |
| Default Language |  |  | ✓ | (3.1.1) Language of Page [WCAG 2.2] |
| Webpage length |  |  | ✓ | Webpage length should be <=14 KB |
| Server status/Availability |  |  | ✓ | Webpage must be available or not down |
| User information |  |  | ✓ | No 'Username' and 'Password' |
| CAPTCH |  |  | ✓ | (1.1.1) Non-text Content [WCAG 2.2] |
| Multiple languages |  |  | ✓ | Webpage should have multiple language version |
| Image ratio |  |  | ✓ | Webpage images number must be <=10 |
| Manual font adjustment option |  |  | ✓ | Webpage should have manual text and font size adjustment option |
| Manual color adjustment option |  |  | ✓ | Webpage color pair should be accessible except [‘red’, ‘green’] and [‘red’, ‘black’] pair/combination |
| Text Font family |  |  | ✓ | Accessible font-family are ‘Tahoma’ **or** ‘Calibri’ **or** ‘Helvetica’ **or** ‘Arial’ **or** ‘Verdana’ **or** ‘Times New Roman’ |
| Text Font size |  |  | ✓ | (1.4.12) Text Spacing [WCAG 2.2] |
| Text pattern |  |  | ✓ | No <*b*> or <*strong*> or <*i*> or <*em*> or <*mark*> or <*sub*> or <*sup*> elements/patterns |
| Content type |  |  | ✓ | Webpage content type should be the combination of **paragraph, image and video** content |
| Number of audio/video content |  |  | ✓ | Webpage number of audios should be >=1 to <=2 and videos should be >=1 to <=2 |

**Appendix C. Webpages in the user study for complexity modeling**

Table C.1 Webpages lists that are used in the user study for validating the proposed WCAEE tool.

**Table 2:** Webpages that are used during proposed tool validation

| **Page ID** | **Page Name** | **Page URL** |
| --- | --- | --- |
| 1 | Eötvös Loránd University | https://www.elte.hu/en/ |
| 2 | [University of Szeged](https://www.google.com/search?sca_esv=392d47dac646a17a&cs=0&sxsrf=ACQVn09QuOXqEwuj3VvfJtDvyf-nVl5KwQ:1709802201140&q=University+of+Szeged&stick=H4sIAAAAAAAAAFVTPY_TMBiW0d3pzi3oFE4MZSmHGFEbx2nSFcTGCdQ75qjOh5PGdtLa157zH5BO_ALEDBszO_0BjAxs_ANG2p6dwva8eZ_n8fPab44Pz08GfDDE_nx8gx6rqu6j_rUolulCFkr3C9HPrwWdLvQa7IgeogIbHEo-mxu8MzDYH-WrhcFBOWNiDeAWL6X2gsgUY8pX_tgq6qa0rlgF7QmYLqPS4CRebU_bicmIksBGCtkcc9OIa5IxzxSolnmemYJS4vuopWGxCK3eDePSNLwca2olKMRetJeQfBi3hcgDG5Ikkg3XoDOgAxehhGWBdR7XpVwZCdYZJ8rSvLEbxdzQkEbCXYP7245LCG-aGkvLdCPsLRtrknla4f0QWEjbGaFVzf6J523uqrtzTHQZ1ttMdo5ZlrcvQutEtTc0GuJ9jthnPJDMutxkM61Kvo214brecE6i2noK1ai2WLgi4L_AN9A5_f3n58PeV_Dh8_cf4AuABy9SqZyPAHYuU3VVXVRJkWnnFjjvATx9XVUyZXqSsqlKk6vKeQSPXgm12UKn24PwePc8dIac6f_yifMWnlyknGxW9k3meBC-rBhLY1VUwnnWewqfDOL2wx2kqXzeLnmRyvMHk7shl6UfujRu7h0fdD8dnr3b_whV1r9sNsLk9gj8BXoiXA81AwAA&sa=X&ved=2ahUKEwigmtLG5eGEAxU-AxAIHZyNDewQ7fAIegUIABDIBA) | https://u-szeged.hu/english |
| 3 | Budapest University of Technology and Economics | https://www.bme.hu/?language=en |
| 4 | University of Pécs | https://international.pte.hu/ |
| 5 | Széchenyi István University | https://admissions.sze.hu/welcome |
| 6 | University of Miskolc | <https://www.uni-miskolc.hu/en> |
| 7 | Semmelweis University | https://semmelweis.hu/english/ |
| 8 | Corvinus University of Budapest | https://www.uni-corvinus.hu/?lang=en |
| 9 | University of Pannonia | https://international.uni-pannon.hu/ |
| 10 | Óbuda University | https://uni-obuda.hu/en/ |
| 11 | [University of Sopron](https://www.google.com/search?sca_esv=392d47dac646a17a&cs=0&sxsrf=ACQVn09QuOXqEwuj3VvfJtDvyf-nVl5KwQ:1709802201140&q=University+of+Sopron&stick=H4sIAAAAAAAAAFVTTW_TMBiW0TZ1aUFTmDiUSxniiNo4TpNeh7gxgbpxjpovJ43tuLHXzvkPSNN-AeIMN87c6Q_gyIEb_4AjbWencHvevM_z-HntN53Ds-MhHY4CskAUPpUVH8DBNSuWaS0KqQYFG-TXDM9qtQY7ogsxQxoHgs4XGiNvMbnR2Bvnq1pjv5wTtgbWFi-Fcv1QFxNMV97EKHhTGlck_fYEhJdhqXESr7an7cTRGEe-ibTLrhsxjzLi6gJykeeZLjCOPA-2NMTqwOidIC51w82RwkYCA-SGe0mUj-K2YLlvQkaJIKM16A7x0IEwIZlvnCe8FCstQSqjkTQ0d-KEMdU0qCBz1uDhtuNEEW0ajoRhOiFyl40xyVwl0X4IxITpjOGKk3_iuZu76u0cE1UGfJvJzDHP8vZFME9ke0PjEdrniD1CfUGMy002V7Kk21gbruOOFlHIjSeTjWyL2mE-_QW-ge7J7z8_H_e_grvP33-AL8A6OE-FtD8Cq3uZyqvqokqKTNm3wP4ArJM3VSVSoqYpmck0uarsJ9bRayY3W2j3-pbV2T0PnkN79r98ar-zji9SGm1W9m1mu5b1qiIkjWVRMftF_7n1bBi3H-4hTsXLdsmLVJw9mt4PuSy9wMFx86Bz0Pt0ePp-_yNU2eCy4nXFbo_AX1Xf6t01AwAA&sa=X&ved=2ahUKEwigmtLG5eGEAxU-AxAIHZyNDewQ7fAIegQIABBA) | https://international.uni-sopron.hu/home |
| 12 | Budapest Business University | https://uni-bge.hu/en |
| 13 | Budapest Metropolitan University | https://www.metubudapest.hu/ |
| 14 | IBS International Business School Budapest | https://www.ibs-b.hu/en/ |
| 15 | Pázmány Péter Catholic University | https://ppke.hu/en |
| 16 | Hungarian University of Fine Arts | https://mke.hu/english/index.php |
| 17 | Moholy-Nagy University of Art and Design | https://mome.hu/en/ |
| 18 | Eötvös József College | https://ejf.hu/en/welcome |
| 19 | University of Kaposvár | https://kc.uni-mate.hu/ |
| 20 | University of Nyíregyháza | https://english.nye.hu/ |
